# Defining the target and the effect of imatinib on the filarial c-Abl homologue

**DOI:** 10.1371/journal.pntd.0005690

**Published:** 2017-07-20

**Authors:** Elise M. O’Connell, Olena Kamenyeva, Sara Lustigman, Aaron Bell, Thomas B. Nutman

**Affiliations:** 1 Laboratory of Parasitic Diseases, Helminth Immunology Section, National Institute of Allergy and Infectious Diseases, Bethesda, Maryland, United States of America; 2 Research Technologies Branch, Biological Imaging Section, National Institute of Allergy and Infectious Diseases, Bethesda, Maryland, United States of America; 3 Laboratory of Molecular Parasitology, Lindsley F. Kimball Research Institute, New York Blood Center, New York City, New York, United States of America; 4 Laboratory of Electron Microscopy, Lindsley F. Kimball Research Institute, New York Blood Center, New York City, New York, United States of America; McGill University, CANADA

## Abstract

**Background:**

Previously we demonstrated the micro- and macrofilaricidal properties of imatinib *in vitro*. Here we use electron and multiphoton microscopy to define the target of imatinib in the adult and microfilarial stages of *Brugia malayi* and assess the effects of pharmacologically relevant levels of imatinib on the adult parasites.

**Methods:**

After fixation of adult *B*. *malayi* males and females, sections were stained with polyclonal rabbit anti-c-Abl antibody (or isotype control) and imaged with multiphoton fluorescent microscopy. Microfilariae were fixed and labeled with rabbit anti-c-Abl IgG primary antibody followed by anti-rabbit gold conjugated secondary antibody and imaged using transmission electron microscopy (TEM; immunoEM). In addition, adult *B*. *malayi* males and females were exposed to 0 or 10μM of imatinib for 7 days following which they were prepared for transmission electron microscopy (TEM) to assess the drug’s effect on filarial ultrastructure.

**Results:**

Fluorescent localization of anti-c-Abl antibody demonstrated widespread uptake in the adult filariae, but the most intense signal was seen in the reproductive organs, muscle, and intestine of both male and female worms. Fluorescence was significantly more intense in the early microfilarial stage (i.e. early morula) compared with later development stages (i.e. pretzel). Anti-c-Abl antibody in the microfilariae localized to the nuclei. Based on TEM assessment following imatinib exposure, imatinib appeared to be detrimental to embryogenesis in the adult female *B*. *malayi*.

**Conclusions:**

At pharmacologically achievable concentrations of imatinib, embryogenesis is impaired and possibly halted in adult filariae. Imatinib is likely a slow microfilaricide due to interference in intra-nuclear processes, which are slowly detrimental to the parasite and not immediately lethal, and thus may be used to lower the levels of *L*. *loa* microfilariae before they are treated within the context of conventional mass drug administration.

## Author summary

While *Loa loa*, or the “African eye worm”, does not commonly cause clinical disease, infection with high blood levels of microfilariae from this helminth infection is problematic in those who receive mass drug administration (MDA) in the eradication efforts of lymphatic filariasis and onchocerciasis. Ivermectin, the drug of choice for both MDA programs, causes a rapid killing of *Loa loa* microfilariae and in those with high pre-treatment levels, a severe inflammatory reaction can result in encephalopathy, and rarely death. Using the filariae *Brugia malayi* as an *in vitro* surrogate model for any of the blood-borne filarial parasites (including *Loa loa*) we have previously shown that imatinib can act as a slow microfilaricide. Understanding imatinib’s targets in adult and microfilariae may predict the *in vivo* effects on *Loa loa* microfilarial loads, and anticipate potential side-effects for future clinical trials. In this study, we localized c-Abl, the target of imatinib, to the reproductive structures of adult *B*. *malayi*, and to the nuclei of the microfilariae. Pharmacologically achievable levels of imatinib most strikingly caused necrosis of developing microfilariae in adult female *B*. *malayi*. These data further support pursuing clinical trials in the safety and efficacy of imatinib for *Loa*. *loa* and other filarial co-infections.

## Introduction

The World Health Organization has prioritized the elimination of lymphatic filariasis and onchocerciasis by 2020, and 2025 respectively through yearly or bi-annual administration of ivermectin or other anthelmintics in filarial-endemic regions of the world, a strategy aimed at interrupting transmission [1]. Despite the significant resources being devoted to this, challenges remain in meeting this goal. In particular, the presence of *Loa loa*, also known as “the African eyeworm”, that is co-endemic with other filariae (e.g. *W*. *bancrofti* or *O*. *volvulus*) in 10 Central African countries has complicated mass drug administration (MDA) programs because of the ivermectin-associated severe/serious adverse events (SAEs [e.g encephalopathy and coma]) [2–4]. While the pathophysiology of these post-ivermectin SAEs is not fully understood, it is believed that rapid killing of the microfilariae (MF) by ivermectin and the inflammatory response induced as a consequence underlies these SAEs [5–7].

Given that severe post-treatment reactions correlate with both the rapidity of MF killing (60–70% killing within 3 days) and the pre-treatment MF levels, [6] a potential alternative approach is to safely lower MF with another agent before treatment with ivermectin. We have recently demonstrated that the tyrosine kinase inhibitor imatinib at pharmacologically achievable concentrations is a slow microfilaricide of *B*. *malayi in vitro* [8].

As efforts are underway to test imatinib on microfilaremic patients we aimed to pursue further understanding in the mechanism of action by which imatinib may be harmful to both the microfilarial and adult stages of blood borne pathogenic filariae. We hypothesized that pharmacologically achievable concentrations of imatinib are able to damage the adult filarial worms as assessed by transmission electron microscopy following drug exposure. We also sought to localize the target of imatinib (c-Abl) in both adult and microfilarial *B*. *malayi* worms using multiphoton fluorescent microscopy and immuno-electron microscopy and to infer its mode of action by assessing the nature of the damage it induces.

## Materials and methods

### Parasite material

Male and female adult *B*. *malayi* worms were obtained under contract from the University of Georgia (Athens, GA) and cleaned as previously described [9]. *In vitro* exposure of parasites to imatinib methanesulfonate salt was performed as previously described for 7 days [8].

*B*. *malayi* crude antigen (BMA) was prepared as previously described [10] from equal number of adult female and adult male worms.

### Immunoblotting

BMA was mixed with Laemmli sample buffer (1610737 Bio-Rad) and 2-mercaptoethanol; 11 μg (lanes 3 and 9) 18.6 μg (lanes 4 and 10) of protein was loaded into a 4–15% precast gel (4561084 Bio-Rad) and transferred onto polyvinylidene difluoride (PVDF) membranes (Bio-Rad) using the Trans Blot Turbo Transfer System (1704150 Bio-Rad). Following blocking with 5% nonfat milk, the membrane was cut in half and incubated with either rabbit anti-c Abl antibody (Ab15130 Abcam) or an isotype control (011-000-003 Jackson Immunoresearch Labs), each diluted to a final concentration of 0.1 μg/mL and incubated overnight at 4^°^C. After washing with TBS, 0.1% Tween-20, the membrane was incubated with HRP-conjugated anti-rabbit IgG (7074 Cell Signaling Technology) at 1:5000 diluted in TBS, 0.1%Tween-20/5% milk at room temperature for 2 hrs. The membrane was washed again with TBS/0.1%Tween-20 and a chemiluminescent substrate was added (1705060 Bio-Rad) and exposed to x-ray film for various times prior to developing.

### Sample preparation and immunostaining:

Approximately 100 adult males or adult females were fixed in 4% paraformaldehyde (15710 Electron Microscopy Sciences, 16% PFA diluted with 1X PBS) at 4^°^C overnight, then placed in 2% agarose in DMEM (1054 Gibco). Sections 200 μm thick were sliced with a Leica VT1000 S Vibrating Blade Microtome (Leica Microsystems) at speed 5, in ice-cold phosphate buffered saline (PBS), and placed in 1% or 2% bovine serum albumin (BSA) in PBS. Sections were permeabilized with 2.5% BSA, 1% Triton-X100 in PBS by agitating slowly at 4^°^C for 1 hour. Blocking was performed with 1% BSA, 10% goat serum and 0.1% Tween-20 in PBS agitating slowly for 1 hour at 4^°^C. Sections were washed with 1% BSA/PBS. Polyclonal rabbit antibody raised against human c-Abl (Ab15130 Abcam) or isotype control rabbit IgG (011-000-003 Jackson Immunoresearch Labs) at a concentration 0.05ug/mL was added to 1% BSA, 1% Triton-X100 in PBS and agitated slowly at 4^°^C for 72–96 hours. Sections were washed twice with 1% BSA/PBS, then counterstained with Alexa Fluor 594 goat anti-rabbit IgG (A31632 Life Technologies) at 1:2000 with 1% Triton-X100, 1%BSA and incubated overnight at 4^°^C agitating. Sections were then washed with 1%BSA/1% Triton/PBS for 16–24 hours agitating at 4^°^C. DAPI (R37606 Molecular probes) was then added. The experiment was repeated 6 times.

### Multiphoton microscopy

Immunostained sections were mounted in 14 mm microwell dishes (MatTek) and imaged using a Leica SP8 inverted 5 channel confocal microscope equipped with dual multiphoton (MP) lasers and a motorized stage. Microscope configuration was set up for three-dimensional analysis (x,y,z) of nematode sections. Mai Tai laser was tuned to 840 nm and InSight Deep See laser to 1150 nm excitation wavelengths. Second harmonic generation (SHG) signal was recorded at 420 nm wavelength. To collect tiled images of a whole section, z stacks consisting of 20 single planes (1 μm each, over a total tissue depth of 20 μm) were acquired and stitched automatically in LAS X (Leica Microsystems) post-acquisition. Images were processed using Imaris (Bitplane) software. Mean intensities of c-Abl fluorescence in various internal structures were analyzed using ImageJ (National Institutes of Health). Statistical analysis was performed using GraphPad Prism 7. Three sections per condition were analyzed.

### Statistical analysis

Statistical analysis of fluorescence intensities was performed using GraphPad Prism 7. Sections from 3 independent experiments were analyzed. Paired Student's t test was used to compare mean fluorescence intensities between structures within one section. Values are presented as means ± SEM as indicated. *, P<0.05; **, P<0.01.

### Electron microscopy

Transmission electron microscopy was performed on imatinib-treated and untreated male and female adult *B*. *malayi*. Day 7 following exposure to 0, 10, 25, or 50 μM of imatinib, worms were fixed in 2% glutaraldehyde and 2.5% paraformaldehyde buffered with 0.1 M sodium cacodylate and 1% sucrose and immediately cut into 3 sections, with the gonad-containing mid-portion further processed for imaging as described elsewhere [11].

For ultrastructure localization of the c-Abl protein in *B*. *malayi* MF, the worms were fixed and processed as described previously [12] using rabbit anti-c-Abl antibody (ABIN753613 Antibodies-Online.com) or rabbit isotype control at a concentration of 1:50. In brief, worms were fixed in 4% paraformaldehyde and 0.1% glutaraldehyde buffered with 0.1 M sodium cacodylate and 1% sucrose. Worms were embedded in LR White resin, sectioned using an ultramicrotome and grids containing the sections were incubated with primary antibody overnight at 4°C. The following day, the grids were washed with buffer, incubated with gold conjugated secondary antibody and contrasted with uranyl acetate before imaging with TEM.

## Results

### Binding of anti-human c-Abl antibody to BMA

To assess the ability of the purified mono-specific antibody raised against the human c-Abl protein to bind specifically to protein(s) in BMA, immunoblotting was performed. As seen (S18 Fig), there was binding of the antibody to a single band of ~ 50kD, whereas the isotype control run at the same dilution failed to react to BMA.

### Localization of the filarial c-Abl tyrosine kinase in filarial adults and MF worms

**Fig 1:**
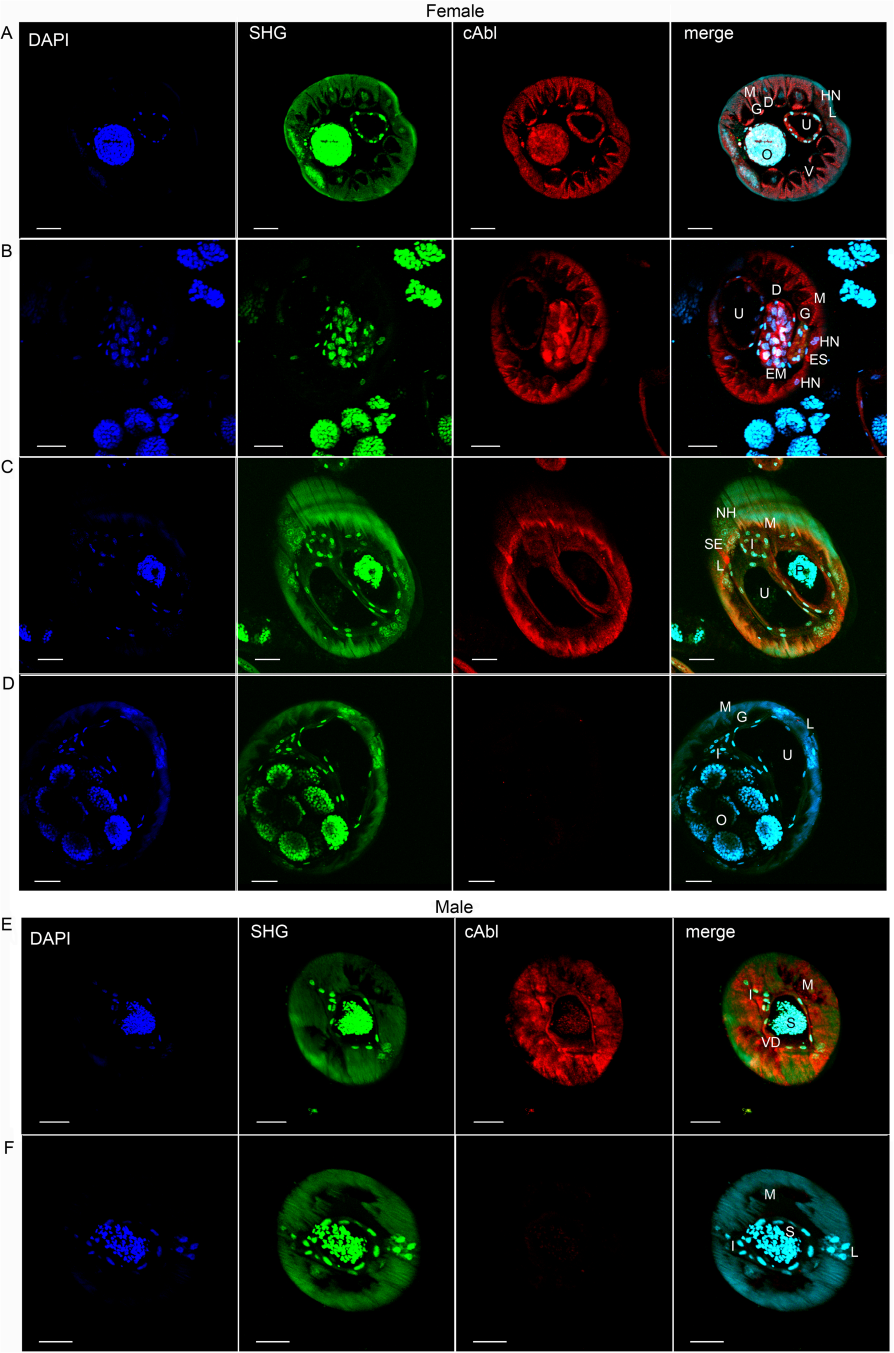
Expression of c-Abl homologue in *B*. *malayi* adults. (*A-C*) Adult females and (*E*) adult males were exposed to polyclonal anti-c-Abl rabbit antibody or (*D*, *F)* isotype control rabbit IgG and counterstained with Alexa Fluor 594 labeled goat anti-rabbit IgG. High levels of expression are seen throughout the hypodermis (including hypodermal cords), gastrointestinal tract, uteri (*A*, female), and vas deferens (*E*, male). Scale bars are 20μm. O, fertilized ova; U, uterus; M, muscle; L, lateral chord; EM, early morula stage; P, early pretzel stage; ES, excretory secretory canal; S, spermatids; VD, Vas Deferens; D, dorsal cord; V, ventral cord; HN, hypodermal nuclei; I, intestine. Images are representative of at least 6 immunofluorescent microscopy experiments.

Using the same highly purified mono-specific antibody raised against the human c-Abl protein we were able to localize the filarial c-Abl-like tyrosine kinase in both adult male and female worms (Fig 1). As can be seen, there was specific staining throughout the hypodermis, including the lateral, ventral and dorsal hypodermal cords, somatic muscle, and reproductive tracts. Within the lateral cords, there was particularly high signal in the excretory-secretory canal (best seen in Fig 1B and 1C). There was variable, but specific staining throughout the intestine (Fig 1).

**Fig 2:**
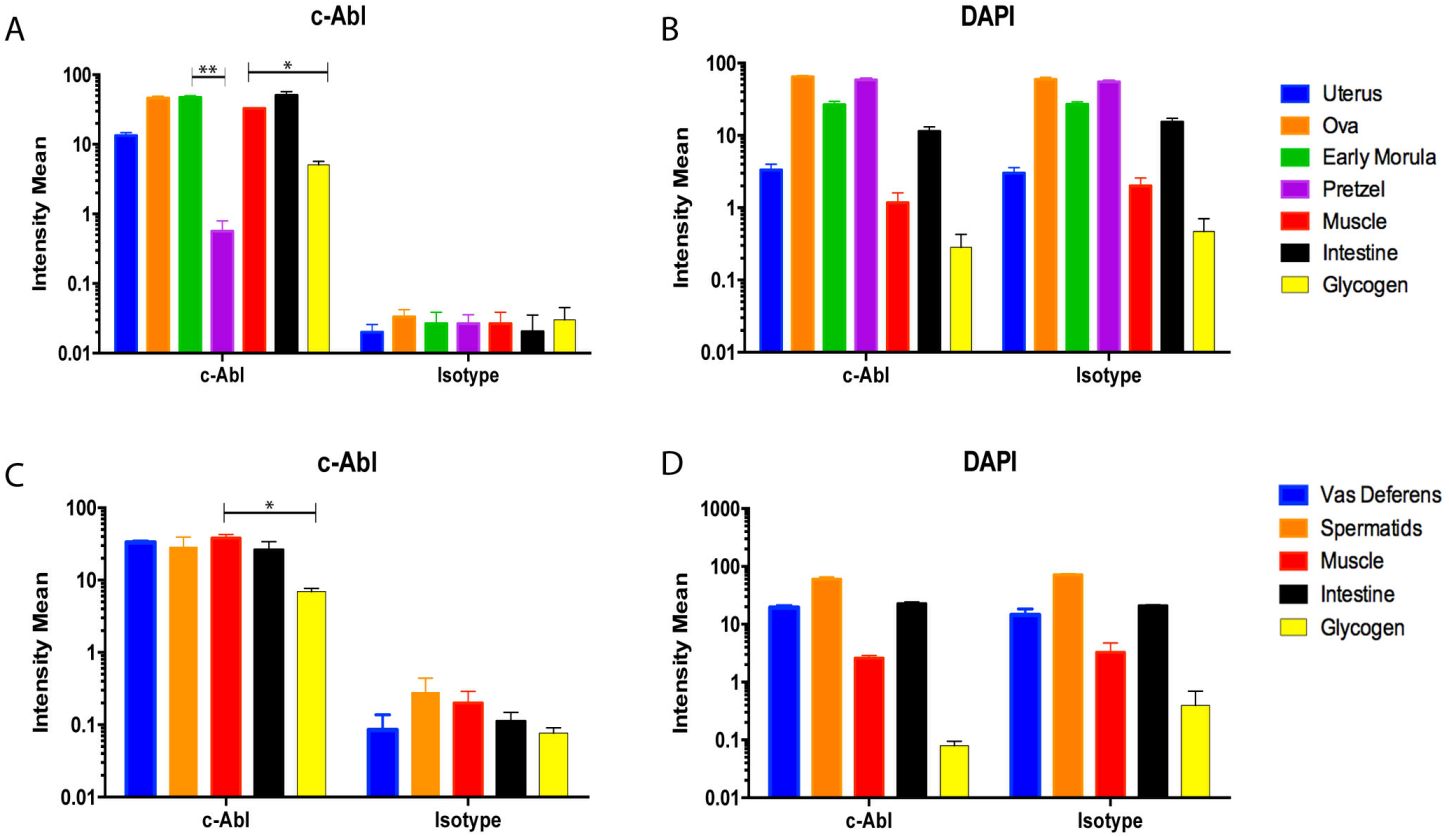
C-Abl expression in developing microfilariae decreases over the course of maturation. C-Abl expression was measured as mean fluorescence intensity in the internal structures of adult worms. (*A*, *C*) The intensity of fluorescence between the reproductive apparatus (*A*, uterus, ovaries, early morula, pretzel; *C*, vas deferens, spermatids), muscle, glycogen, and intestine is significantly more in the structures treated with anti-c-Abl antibody compared with isotype control. (*A*) Adult females show over the course of embryonic development a decrease in c-Abl expression from the early morula to the pretzel stage (p = 0.0035). Higher c-Abl expression is also seen in muscle compared with glycogen in females (*A*, p = 0.0187) as well as males (*C*, p = 0.0155). (*B*, *D*) Fluorescence intensity generated by the nuclear stain DAPI, is essentially identical between the c-Abl and isotype control conditions. Three sections per condition were analyzed, and the experiment was repeated 3 times; means ± SEM.

Quantitative analysis of mean fluorescence intensities detected in internal structures (S1 Fig) has confirmed significant differences in the amount of filarial c-Abl-like tyrosine kinase expression in various organs of adult worms (Fig 2).

In the females, it is notable that among the early stages of embryonic development, (Fig 1A and 1B) c-Abl expression is seen throughout the interstitium surrounding the developing embryo, as well as in the ovary and uterine lining. However, over the course of microfilarial development the expression of c-Abl appears to decrease in the interstitium surrounding the developing worm, and by later development (pretzel stage, Fig 1C), c-Abl expression surrounding the developing embryo is minimal. This signal change is further quantified in Fig 2, where the early morula stage has higher c-Abl expression compared with the pretzel stage (p = 0.0035).

**Fig 3:**
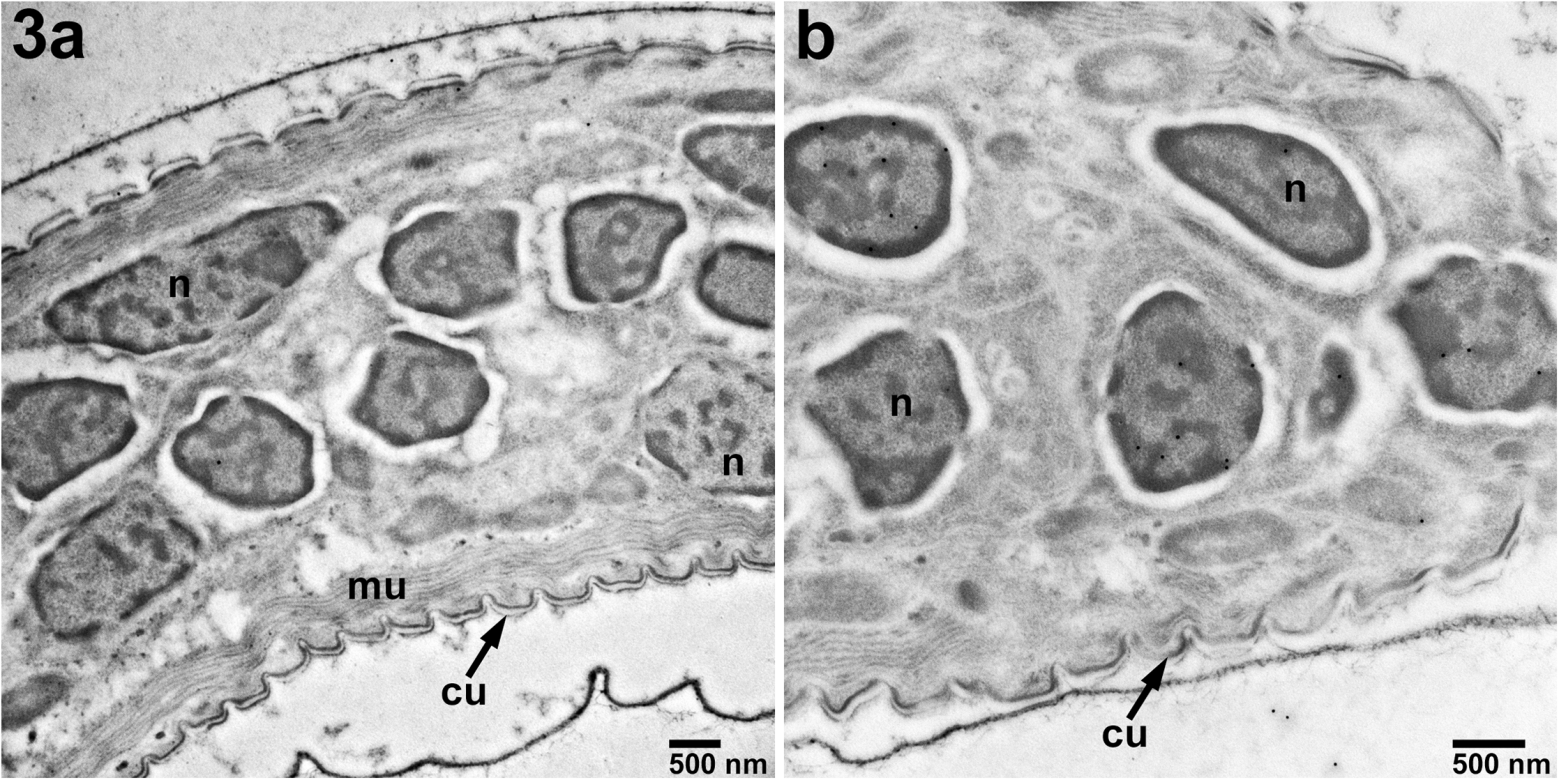
Localization of c-Abl in *B*. *malayi* microfilariae by immune-transmission electron microscopy. A)Staining with isotype control antibody (18,500X). B)Localization of c-Abl-like protein in the nuclei of MF (black dots, 13,000X). Magnification: bar 500 nm. n—nucleus, mu–muscle, cu—cuticle.

When the anti-c-Abl primary antibody was used in immunoEM of *B*. *malayi* microfilariae the protein was localized solely to the MF nuclei (Fig 3B) and not to any other specific structures within the microfilariae. As expected, isotype control antibody exhibited very little staining of microfilariae (Fig 3A).

### Defining the effects of imatinib on filarial parasites

**Fig 4:**
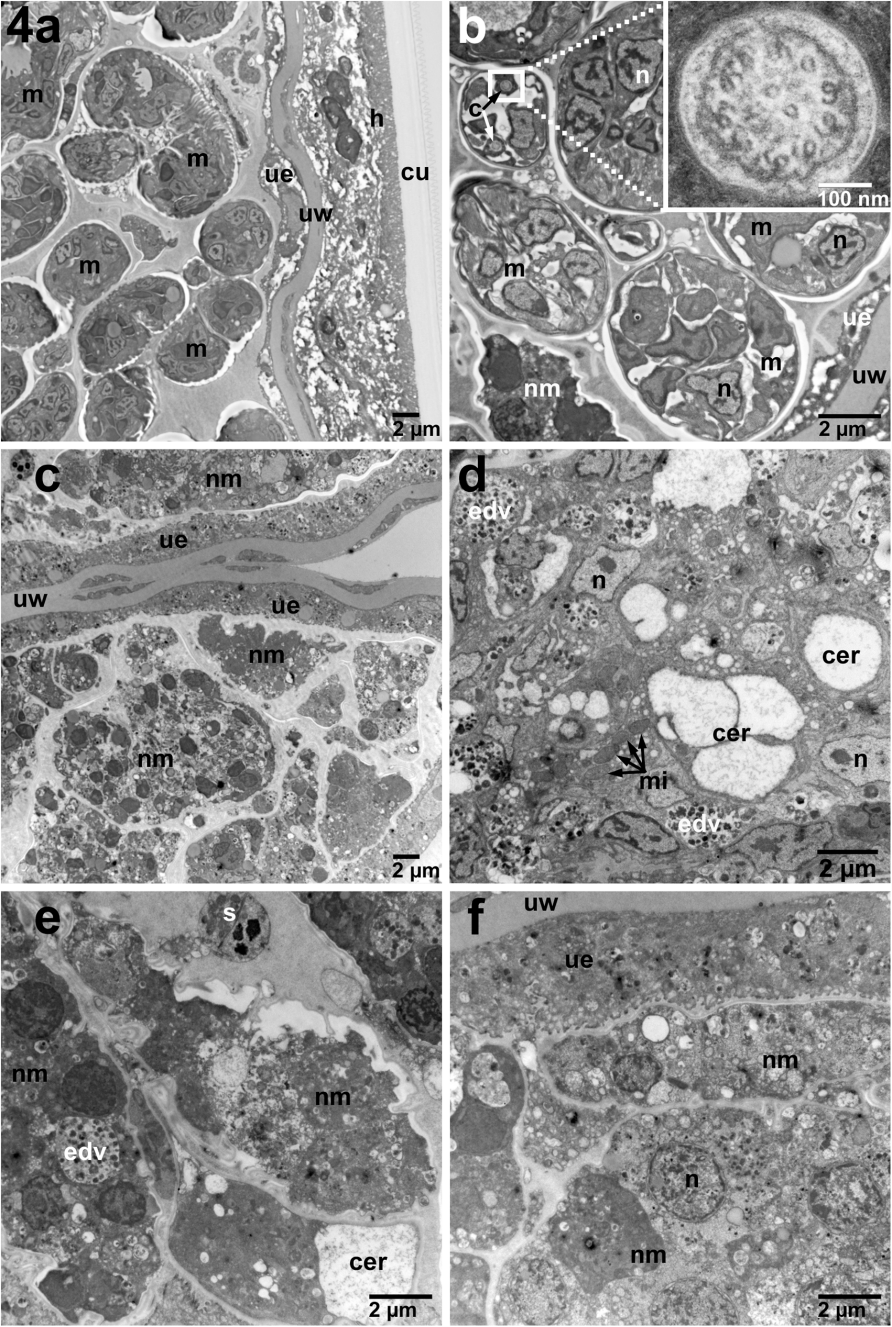
Changes in adult female *B*. *malayi* treated for 7 days with 10 μM imatinib compared with untreated cultured control worms. A) Low power (2,900X) demonstrating overall significant necrosis of developing microfilariae (C) compared with the untreated females (A). B) Higher magnification of females (6,800X) shows treated females (D-F) have numerous electron dense vacuoles (EDV), distended cisternae of the endoplasmic reticulum (CER), distorted microfilariae morphology and a spectrum of microfilarial necrosis compared with untreated (B) worms. Inset of cilium is 68,000X. m–Developing microfilariae, ue–Uterine epithelium, uw–Uterine Wall, h–Hypodermis of the lateral cord, cu–Cuticle, nm–Necrotic Microfilaria, n–Nucleus, s–Spermatocyte, mi–Mitochondria, c–Cilium, bi–Basal infoldings, b–Basement membrane, g–Glycogen, nc–Nerve Cord, w–*Wolbachia*, edv–Electron Dense Vacuoles, cer-cisternae of endoplasmic reticulum.

**Fig 5:**
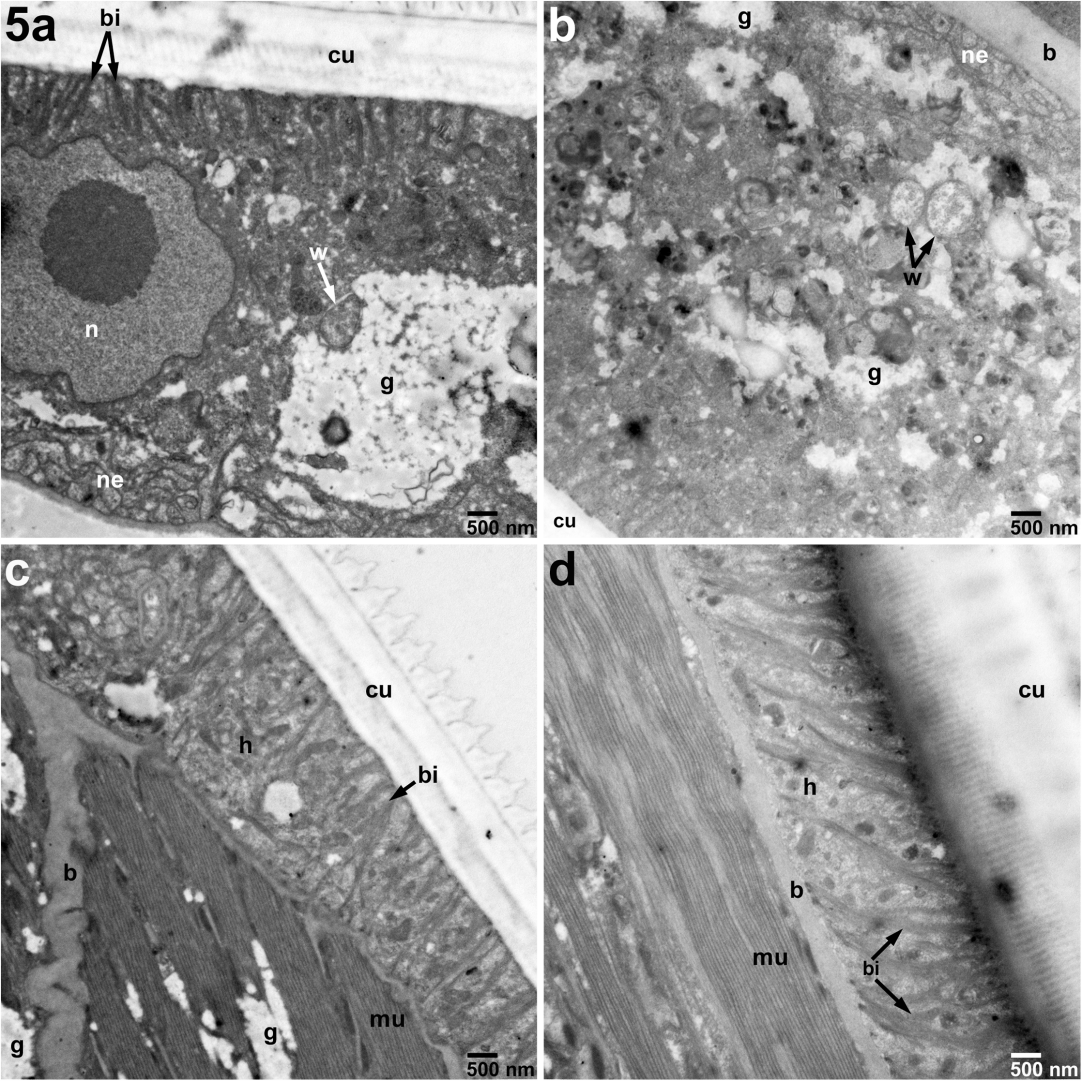
Hypodermis unchanged in adult female *B*. *malayi* treated for 7 days with 10 μM imatinib compared with untreated cultured control worms. A) Untreated hypodermis *Wolbachia* (11,000X) appear similar to B) *Wolbachia* (W, arrows) from treated females. C) Untreated female cuticle, muscle, and hypodermis appear similar to D) treated the treated females (11,000X). h–Hypodermis of the lateral cord, cu–Cuticle, n–Nucleus, bi–Basal infoldings, mu–Muscle, b–Basement membrane, g–Glycogen, nc–Nerve Cord, w–*Wolbachia*.

**Fig 6:**
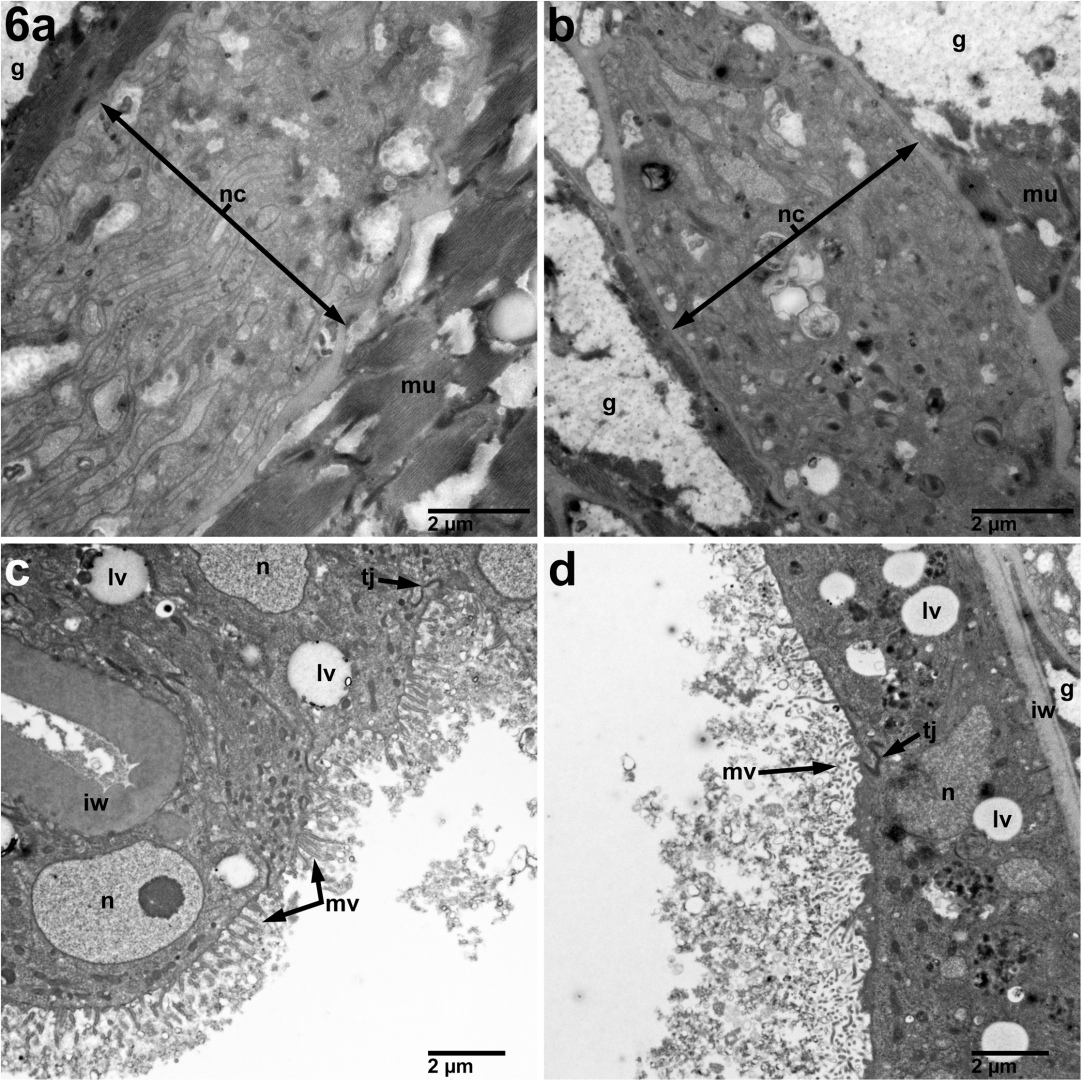
Nerve and intestine unchanged in adult female *B*. *malayi* treated for 7 days with 10 μM imatinib compared with untreated cultured control worms. B) Nerve chord of treated adult female (9,300X) without a significant difference as compared to controls (A). D) No change in the intestine morphology (6,800X) compared with untreated controls (C). n–Nucleus, bi–Basal infoldings, mu–Muscle, b–Basement membrane, g–Glycogen, nc–Nerve Cord, mi–Mitochondria, mv–Microvilli, tj–Tight Junction, lv–Lipid Vacuole, iw–Intestinal Wall.

Having identified where in the parasite the c-Abl homologue is expressed, we next wanted to determine how imatinib exploits these anatomical niches to harm or kill the parasites. Thus, using TEM of sections prepared from adult *B*. *malayi* parasites exposed to 10 μM imatinib we were able to demonstrate significant damage to the reproductive apparatus in female worms (Fig 4). The most striking feature was the significant necrosis of developing microfilariae. In the reproductive tracts of females treated with imatinib, some structures of the MF, such as the endoplasmic reticulum, were clearly distorted (Fig 4B). However, most other structures in the developing microfilariae were completely unrecognizable, with only clusters of electron dense vacuoles found throughout the bodies of individual MF surrounding remnants of nuclei (Fig 4 panels A2 and B2-B4). Interestingly, there were no significant changes to *Wolbachia* at 10 μM (Fig 5A and 5B), nor at higher concentrations of imatinib (S7). At 10 μM, the hypodermis (Fig 5C and 5D), muscle (Fig 5C and 5D), nerve chords (Fig 6A and 6B), and intestine (Fig 6C and 6D) in treated females showed minimal or no morphologic difference compared to controls.

**Fig 7:**
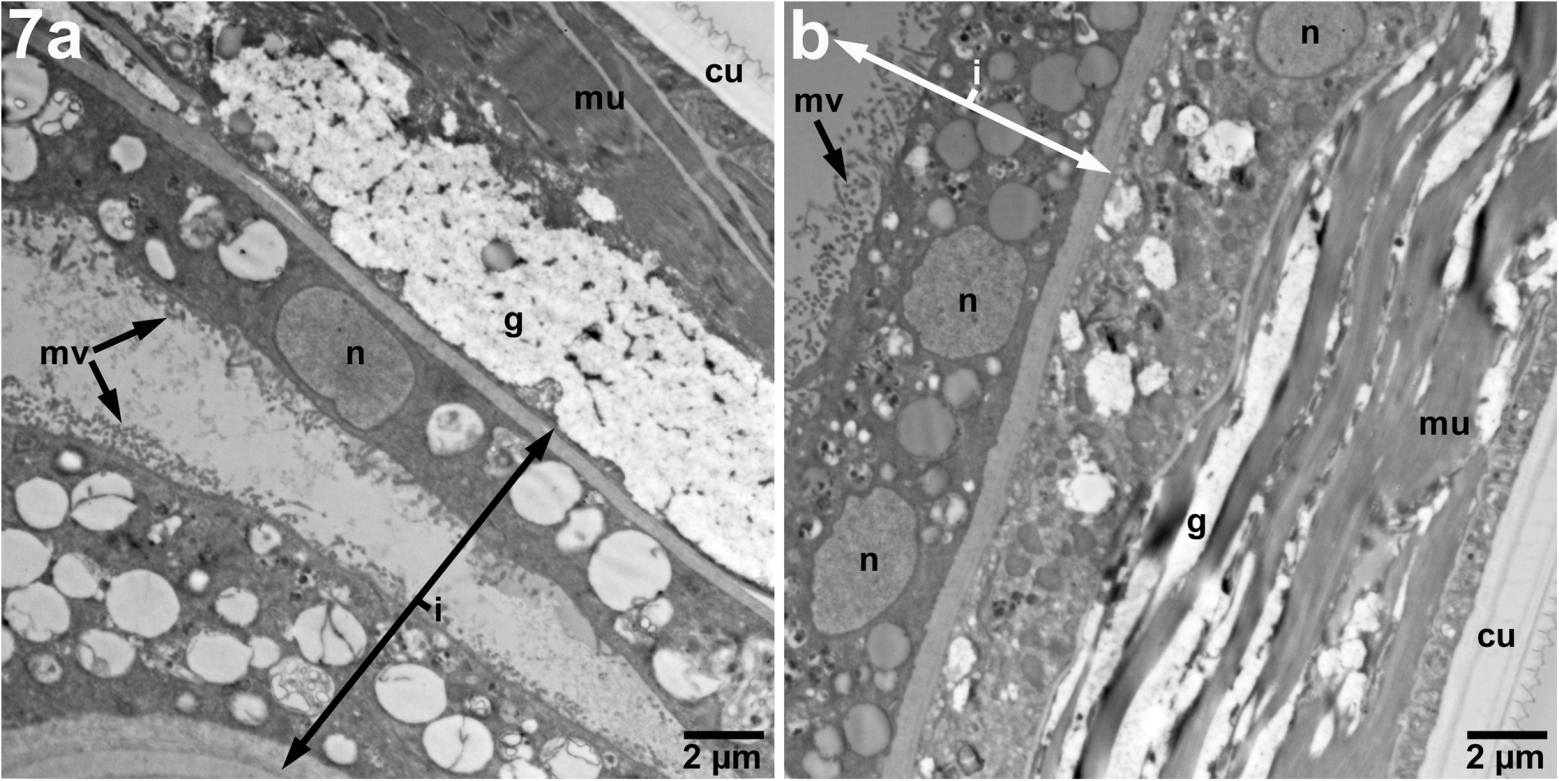
Imatinib-treated adult male *B*. *malayi* do not show significant alterations at pharmacologically achievable dosing. Adult males (4,800X) exposed to imatinib at 10 μM demonstrate minimal disorganization of glycogen and muscle compared with controls. uw–uterine wall, n–hypodermal nuclei, g–glycogen, mu–muscle, cu–cuticle, i–intestine.

Adult males treated with 10 μM of imatinib showed minimal disorganization of the somatic muscle in some areas (Fig 7), however, overall there were no striking differences in features at this drug concentration compared with control males.

Doses higher than 10 μM were substantially more macrofilaricidal *in vitro*. As can be seen in Supplemental images (S5 Fig in comparison to S2–S4 Figs), at 25 μM all developing microfilarial structures are destroyed, which is similarly seen at higher doses (S8 and S9 Figs). At 50 μM, both males and females showed significant architecture distortion of the hypodermis, and the nerve contained within the hypodermal cords also appeared obviously damaged (S7, S11, S15 and S16 Figs).

## Discussion

Imatinib mesylate (Gleevec) is an orally administered small molecule c-Abl (and other tyrosine kinase) inhibitor with over 15 years of safety and pharmacokinetic data. Initially designed to target the constitutively active Bcr-Abl1 tyrosine kinase seen in chronic myelogenous leukemia (CML), the pharmacokinetics (PK) have been described in healthy volunteers, patients with CML as well as other malignancies (gastrointestinal stromal tumor, glioma)[13, 14]. Following oral administration, C_max_ is achieved after 2–4 hours, with 98% bioavailability, and is highly protein bound[13–15]. The half-life of the imatinib parent compound is 18 hours, and 40 hours for its primary active metabolite[13, 14]. There is some inter-patient drug level variability, thought to be due to differences in drug metabolism by cytochrome-P450 isoform polymorphisms or efflux pumps,[14] although there has been conflicting data in this respect [16]. There is also evidence that blood concentrations of drug do not necessarily mirror intracellular levels [17]. No studies have assessed drug pharmacokinetics on the Central African population, although one study has evaluated genetic polymorphisms and the influence on imatinib blood levels in a West African population. It found that Nigerians were more likely to have genotypes that were associated with lower trough drug levels, however even between ethnic tribes allelic frequencies were significantly different [18]. Thus, it is clear that in areas endemic to *Loa loa* infection in Central Africa that more work still needs to be done in assessing imatinib pharmacokinetics in this population.

In general, imatinib is extremely well-tolerated. With chronic daily use, common side effects of imatinib include edema, gastrointestinal complaints, fatigue, rash, and headache[19]. Infrequently are cytopenias seen outside the setting of hematologic malignancy [19]. In a recent large series examining 10 years of safety data prospectively gathered on CML patients, only 6.9% of subjects discontinued treatment due to adverse events, and no new adverse events were discovered due to long term exposure of the drug [20]. The targeted mechanism of action of the drug, and the fact that it does not act by DNA alteration likely account for these findings. However, with a one-time dose of imatinib, which is what we would propose for filarial treatment, side effects most likely to be encountered would more accurately be reflected in the data from the pharmacokinetic studies evaluating a single dose of drug in healthy subjects. In these studies, a minority of subjects developed headaches or nausea, and none developed any other significant adverse events [21, 22].

We have previously shown that the filarial homologue of the human c-Abl protein is highly expressed in adult *B*. *malayi*¸ and that imatinib likely acts by inhibition of the filarial protein. Here we have shown that its inhibition *in vitro* in worms following exposure to 10 μM of imatinib causes physical damage to structures crucial for reproduction in adult female *B*. *malayi*. Following 600 mg of imatinib, a dose corresponding to that used in gastrointestinal stromal tumors, an average blood concentration of 13.2 μM (95% CI ± 6.4 μM) is achieved [23, 17].

We have used *B*. *malayi* given that these are the only human pathogenic filariae that can be obtained in large quantities from suitable small animal models. *B*. *malayi*, like the other pathogenic blood borne filariae including *L*. *loa*, has sheathed microfilariae, and each life stage has significant structural similarity [24–26]. We have previously reported the high degree of genetic identity in the c-Abl protein sequence between *L*. *loa* and *B*. *malayi*. Moreover, the predicted protein-drug interaction of the *L*. *loa* c-Abl homologue and imatinib, a small molecule inhibitor of c-Abl, [8] is largely identical to the site of interaction between the *B*. *malayi* c-Abl homologue and imatinib. Our previous study also demonstrated that at pharmacologically achievable concentrations (5 μM and 10 μM) imatinib acted as a slow (<50% killing by 4 days) microfilaricide in *B*. *malayi in vitro*. Slow killing is believed to be an important characteristic of a drug to be used as a microfilaricide in *L*. *loa*, as it is thought that rapid antigen release in the course of a high burden of microfilarial death is what causes adverse treatment reactions [6].

In *B*. *malayi* microfilariae, the c-Abl homologue was localized to the somatic nuclei (Fig 3). Over the course of development, these cells coalesce into syncytia called the hypodermis in adult filariae [27]. In nematodes, the hypodermis is responsible for cuticle integrity [28] and many metabolic processes, as it contains fat, glycogen, nerves, mitochondria, and endoplasmic reticulum and is a major site for carbohydrate metabolism [27, 29, 30]. The hypodermis separates the cuticle from the somatic muscle, and, in four places, it bulges into two lateral cords, one dorsal and one ventral cord (together called “hypodermal cords”). Each lateral cord contains an excretory secretory canal, the endosymbiont *Wolbachia*, nerve bundles, and hypodermal nuclei [27]. We have demonstrated that the protein expression of the c-Abl homologue is consistent in the anatomical structures associated with the hypodermis from microfilariae to adult stages.

Similar to the hypodermis, the female uterus is also highly metabolically active, and is also the site of a large amount of diverse protein transcription to support the developing embryos [31]. Direct evidence for the involvement of the filarial c-Abl homologue in reproduction is demonstrated by examining the impact of 10 μM imatinib on embryogenesis in the female. TEM images demonstrated necrotic MF (Fig 4A and 4B) compared with untreated controls. The localization of c-Abl to the uterine epithelium supports the idea that c-Abl is involved in processes necessary for MF development.

Filarial embryonic development proceeds linearly down two parallel uteri until mature sheathed and elongated microfilariae are expelled from the vagina. After multiple divisions of the fertilized zygote, the eggshell, which will ultimately become the microfilarial sheath, separates from outer layer of the embryo, soon followed by the eggshell becoming intricately folded (best seen in Fig 3A control) [32]. At this development stage within the uterus and distally to the vulva the uterus is lined with large apocrine cells [32]. Just as the composition of the uterine epithelium is different at various embryonic stages, c-Abl localization around the embryos appears to change and decrease as the developing microfilariae mature and eventually elongate. Early in development, fluorescence is measured throughout the fertilized ova (Figs 1A and 2). As the embryo matures, the specific staining consolidates around the outer surface, as can be seen in the morula stage (Fig 1B), however the overall fluorescence is similar to that seen in the fertilized ova (Fig 2). Finally, as can be seen in the young microfilarial pretzel stage, the staining is limited to only the uterine lining (Fig 1C), and the signal is significantly less than earlier stages (Fig 2). Interestingly, these changes mimic what was seen in a previous study examining the localization of the microfilarial sheath protein 2 [33]. Additionally, the effect of imatinib on developing microfilariae is similar to effects observed on the reproductive apparatus of *Schistosoma mansoni* [34], and in *Echinococcus multilocularis* [35]. One unifying observation made in the localization of c-Abl to the hypodermis and uterine lining and the destruction in these areas seen at very high imatinib concentrations (S7, S10, S11 and S14 Figs) in both of these areas is that significant glucose metabolism takes place in the hypodermis and uterine epithelium [36]. We therefore hypothesize that the c-Abl homologue may be involved in maintenance of the developing embryos’ outer eggshell and/or in glucose utilization related to supplying energy for this process. Interruption of this protective layer may ultimately lead to the microfilarial necrosis seen in utero in imatinib-treated females.

Given the genetic similarities between *B*. *malayi* and *L*. *loa*, the above offers evidence that a single oral dose of imatinib may not only affect circulating *L*. *loa* microfilariae but may also impair embryogenesis, and potentially future fecundity in adult *L*. *loa* filariae. In an effort to avoid the deleterious effects of ivermectin administration in Loa-endemic areas a strategy termed Test (and) Not Treat (TNT) has been suggested whereby screening the entire “at risk” population is performed [37] and treatment with ivermectin in only those with levels of *L*. *loa* below a certain threshold. However, left unaddressed is what to do with those people with levels of microfilariae above this “safe” threshold. Moreover, given that for some other blood-borne filariae (e.g. *M*. *perstans*) there is a positive correlation between the intensity of *M*. *perstans* and that of *L*. *loa*, [38] not treating those with the highest *L*. *loa* levels may have implications for other co-incident filarial infections as well. Thus, if the TNT strategy is to be adopted, then the dosing of imatinib in those excluded individuals may be a safe and already available treatment. Clinical trials of imatinib in Loa-endemic areas to assess its efficacy and safety in reducing blood microfilarial levels are of utmost importance.

## Supporting information

S1 Fig Identification of *B. malayi* structures expressing c-abl homologue. Internal structures were identified as regions of interest (dotted white lines) based on c-abl and DAPI fluorescence. Early morula (*A*), four ovaries (*B*), and uteri (*C*) in female sections are indicated with white dotted lines and white arrows. Scale bars are 20 μm. Representative of 6 imaging sessions. Related to Figs 1 and 2.
(TIF)

S2 Fig 10μM imatinib exposure during early *B. malayi* embryonic development. No comparison control.
(TIF)

S3 Fig Morphology of in utero *B. malayi* microfilaria following exposure to 10μM imatinib and control. An area of preserved architecture in the treated microfilariae.
(TIF)

S4 Fig Necrosed in utero *B. malayi* microfilaria following exposure to 10μM imatinib. Significant distortion beyond recognition of most organelles.
(TIF)

S5 Fig Developing *B. malayi* microfilaria following exposure to 25μM imatinib and control. Damage beyond recognition of internal organelles of developing microfilariae in treatment group with all microfilariae being homogenously affected.
(TIF)

S6 Fig Female muscle and glycogen structures following imatinib 25μM and control. No definitive difference between treated and untreated worms.
(TIF)

S7 Fig Female hypodermis following imatinib 50μM and control. Diminished glycogen and distorted architecture in the treated worm.
(TIF)

S8 Fig Developing microfilariae post exposure to imatinib 50μM and control. Damage beyond recognition of internal organelles of developing microfilariae in treatment group with all microfilariae being homogenously affected.
(TIF)

S9 Fig Developing microfilariae post exposure to imatinib 50μM and control, high power. Internal structures of treated group not identifiable.
(TIF)

S10 Fig Female muscle and glycogen structures following imatinib 50μM and control. No significant changes.
(TIF)

S11 Fig Female nerve cord following imatinib 50μM and control. Treated worm with significant nerve damage.
(TIF)

S12 Fig Male hypodermis following imatinib 25μM and control. No obvious changes.
(TIF)

S13 Fig Male muscle and glycogen structures following imatinib 50μM and control. No definitive alterations of the muscle/glycogen organization.
(TIF)

S14 Fig Male muscle and glycogen structures following imatinib 50μM and control, higher power. No definitive alterations of the muscle/glycogen organization.
(TIF)

S15 Fig Male hypodermis following imatinib 50μM and control. Alteration of hypodermal structures seen in treated worm.
(TIF)

S16 Fig Male nerve cord following imatinib 50μM and control. Treated worm with significant nerve damage.
(TIF)

S17 Fig Male vas deferens following imatinib 50μM and control. No clear changes.
(TIF)

S18 Fig Immunoblot of *B. malayi* antigen (lanes 3, 4, 9,10) exposed to anti-c abl antibody (lanes 1–6) or isotype control antibody (lanes 7–12). While no bands are present in the isotope control lanes, a single discrete band is observed in lanes 3 and 4, detected by the anti-c abl antibody and approximately 50kD in size. Molecular markers are in lanes 1 and 12.
(TIF)
