# Supplementary figures and images for: Defining the target and the effect of imatinib on the filarial c-Abl homologue
Source: PLoS Negl Trop Dis. 2017 Jul 20;11(7):e0005690. doi: 10.1371/journal.pntd.0005690 (PMC5538754; doi:10.1371/journal.pntd.0005690)

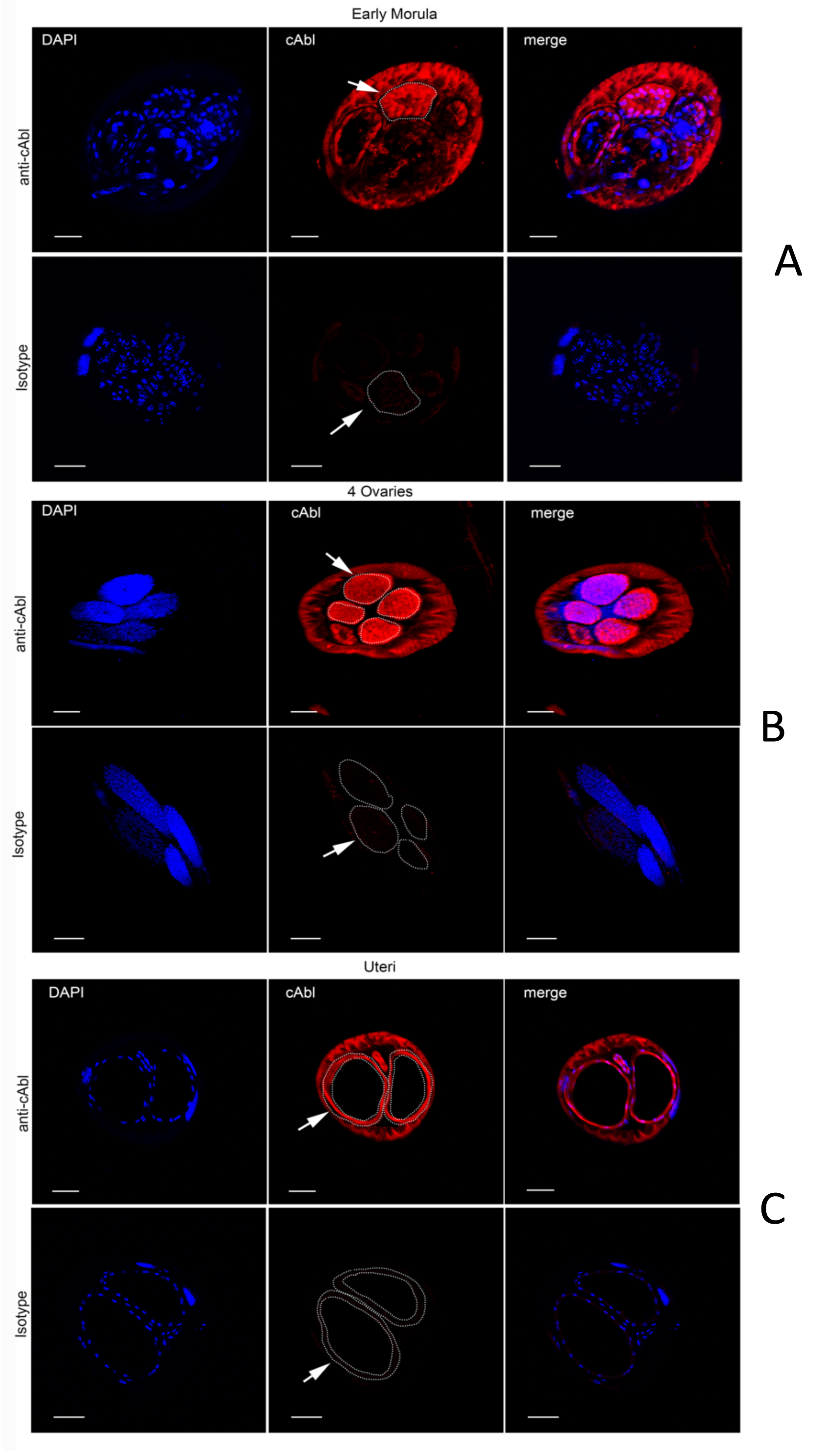

Supplement: S1 Fig — Internal structures were identified as regions of interest (dotted white lines) based on c-abl and DAPI fluorescence. Early morula (A), four ovaries (B), and uteri (C) in female sections are indicated with white dotted lines and white arrows. Scale bars are 20 μm. Representative of 6 imaging sessions. Related to Figs 1 and 2. (TIF) [file pntd.0005690.s001.tif]

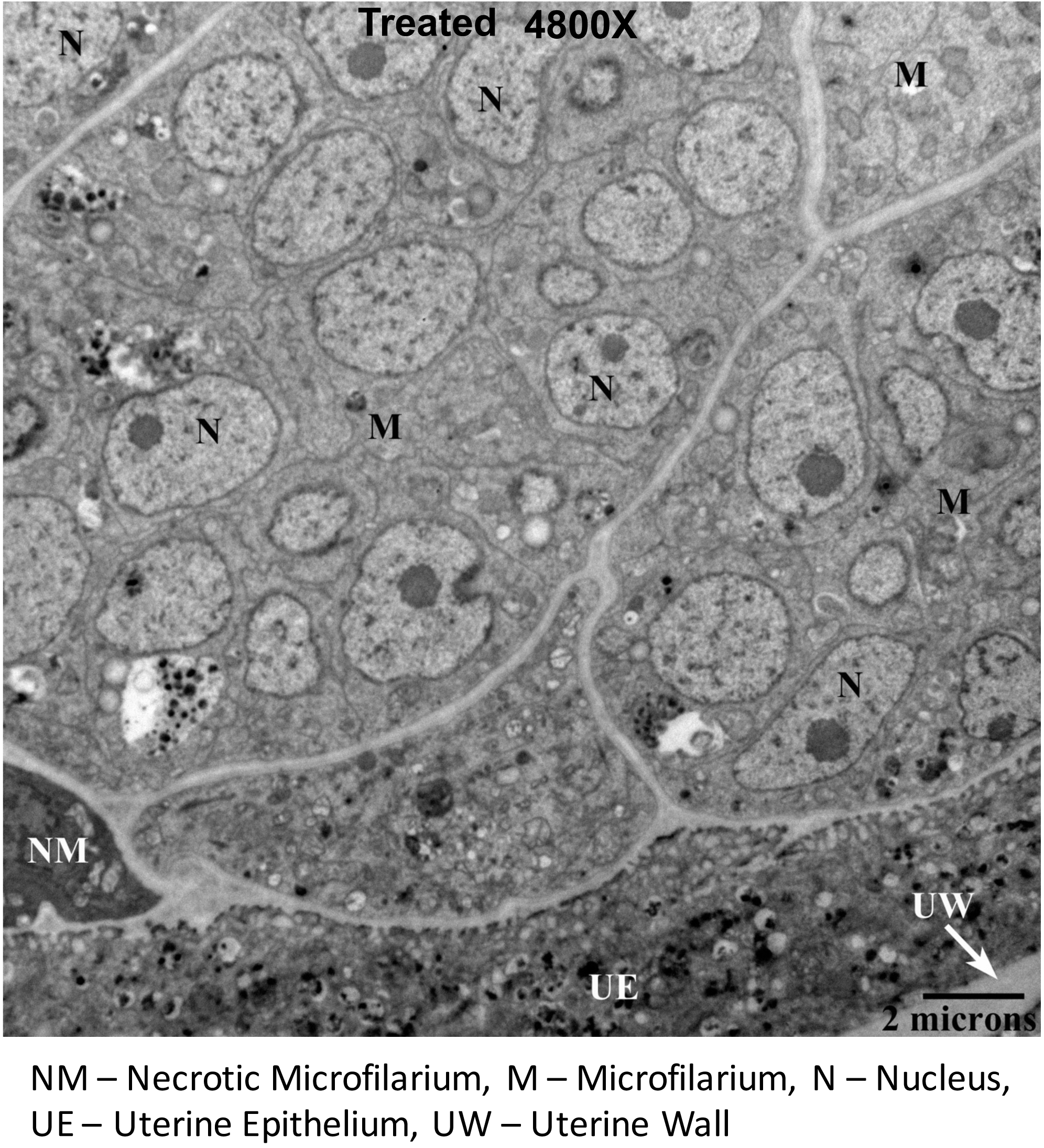

Supplement: S2 Fig — No comparison control. (TIF) [file pntd.0005690.s002.tif]

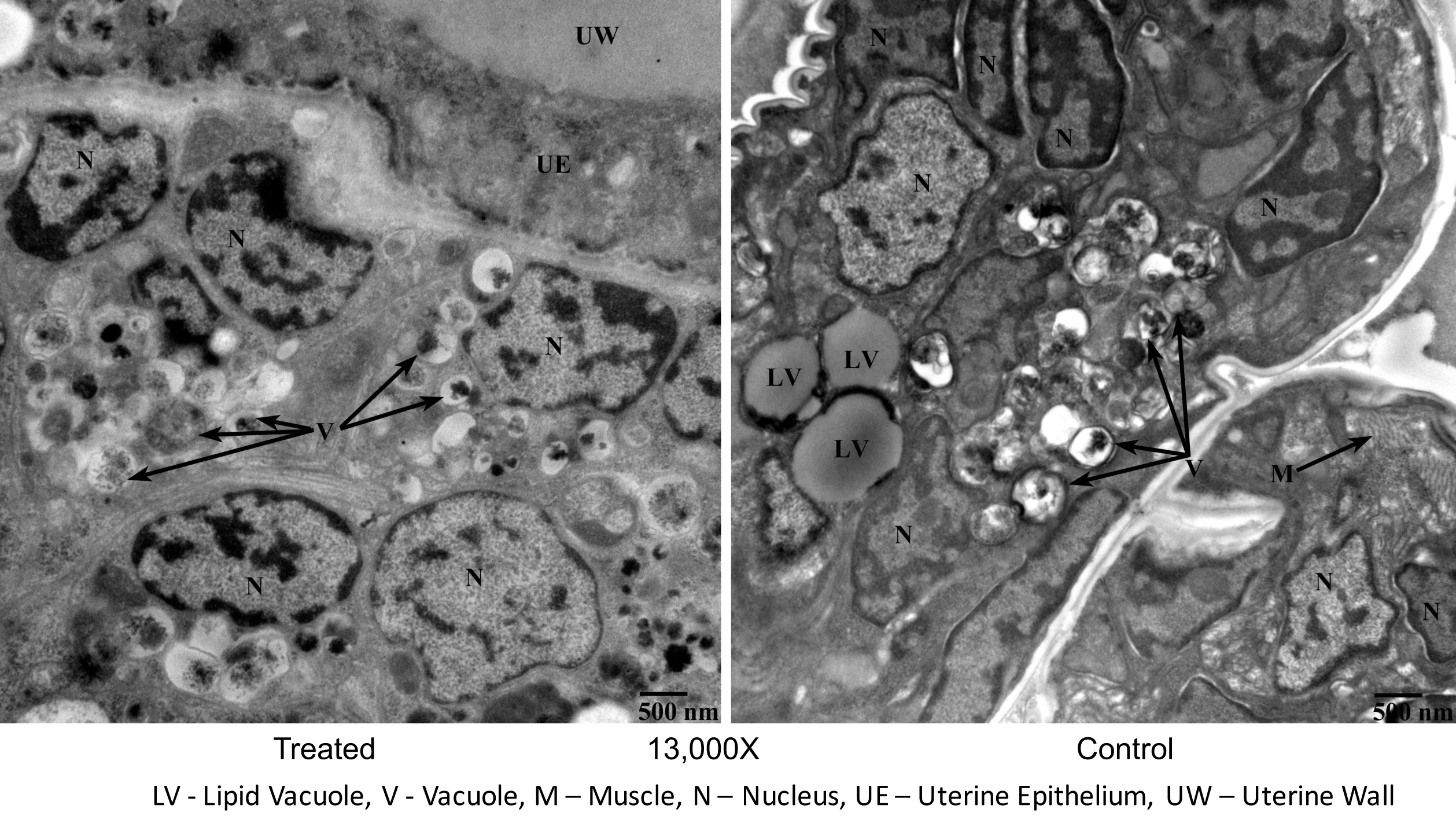

Supplement: S3 Fig — An area of preserved architecture in the treated microfilariae. (TIF) [file pntd.0005690.s003.tif]

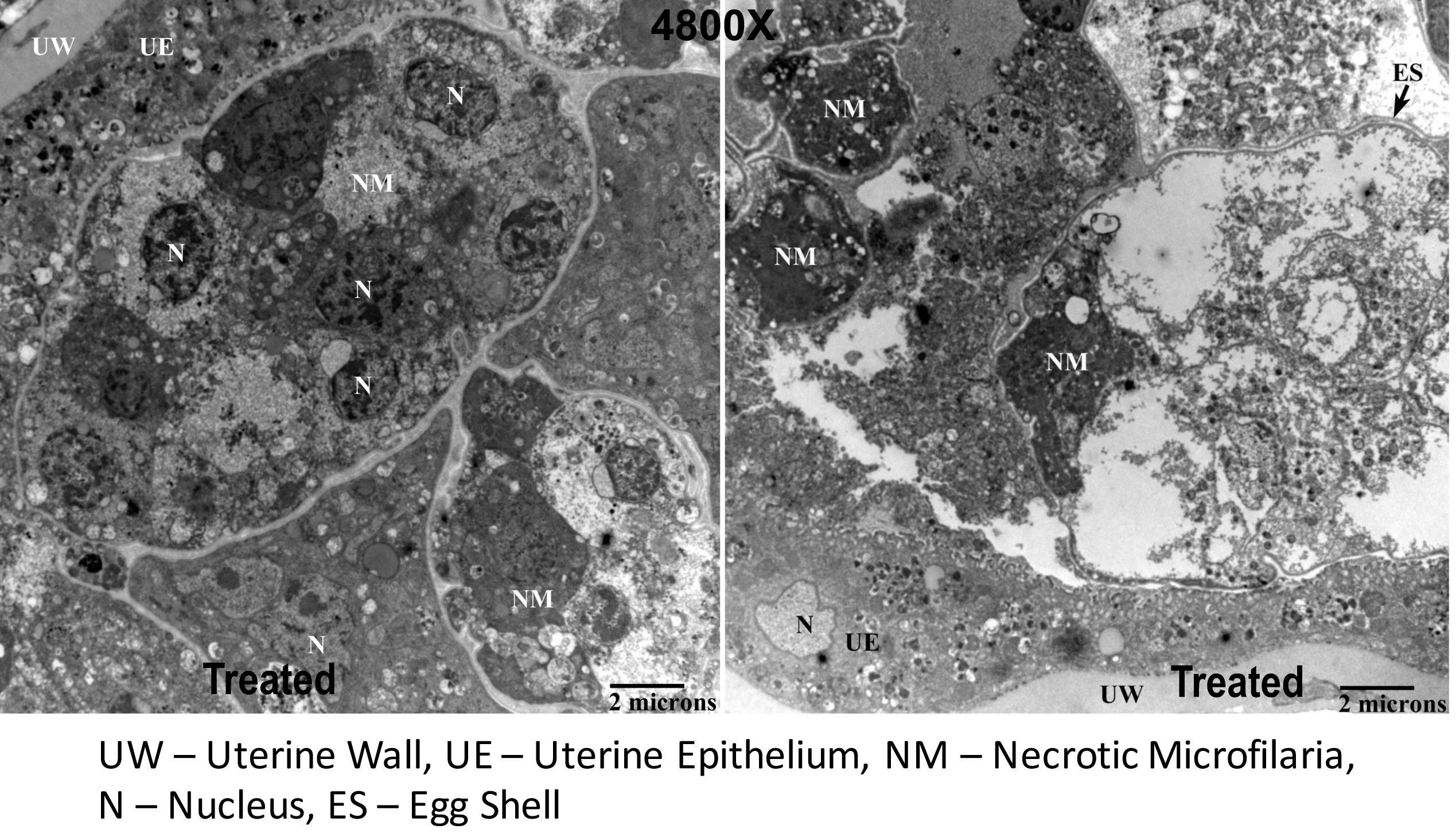

Supplement: S4 Fig — Significant distortion beyond recognition of most organelles. (TIF) [file pntd.0005690.s004.tif]

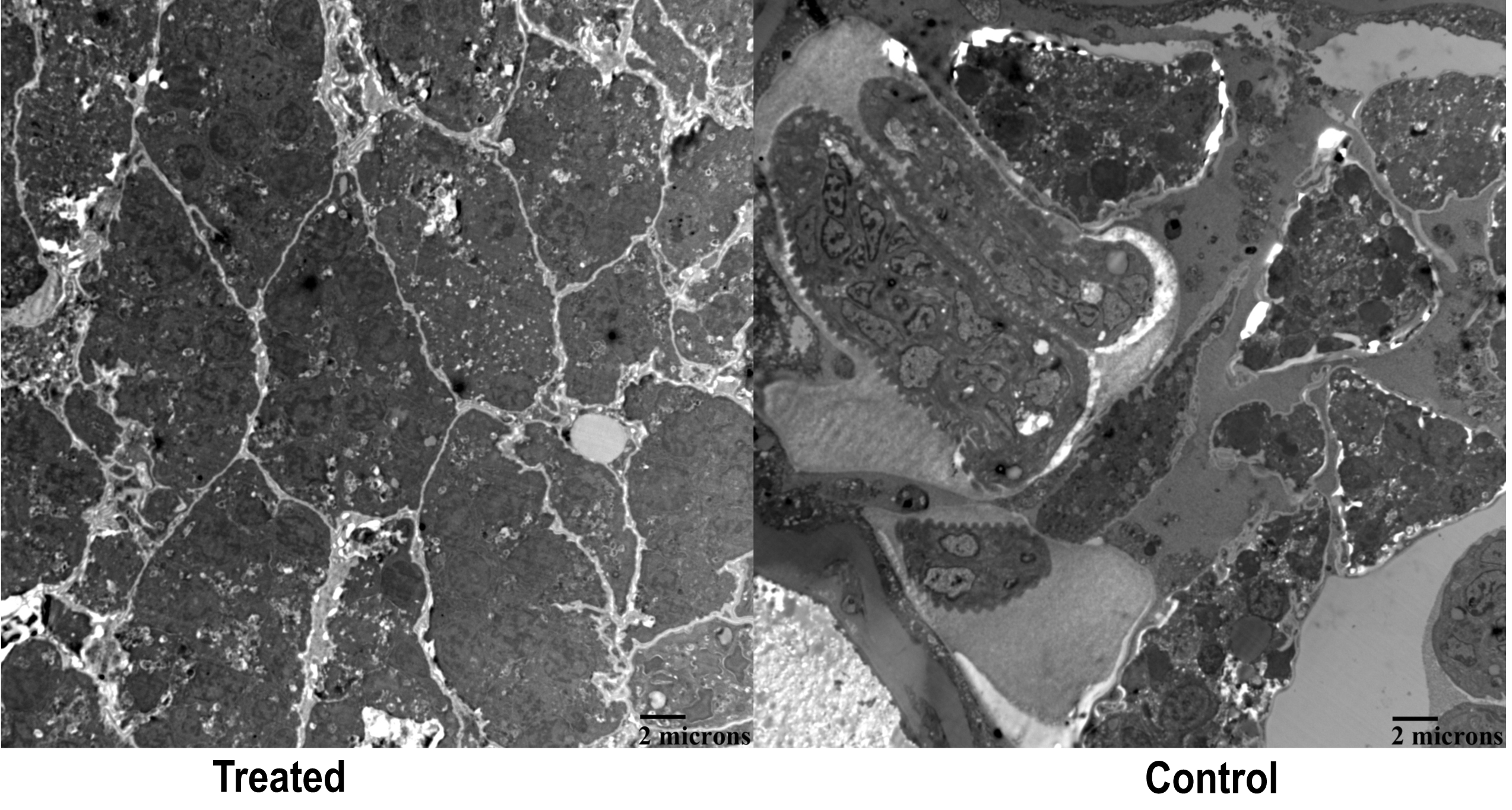

Supplement: S5 Fig — Damage beyond recognition of internal organelles of developing microfilariae in treatment group with all microfilariae being homogenously affected. (TIF) [file pntd.0005690.s005.tif]

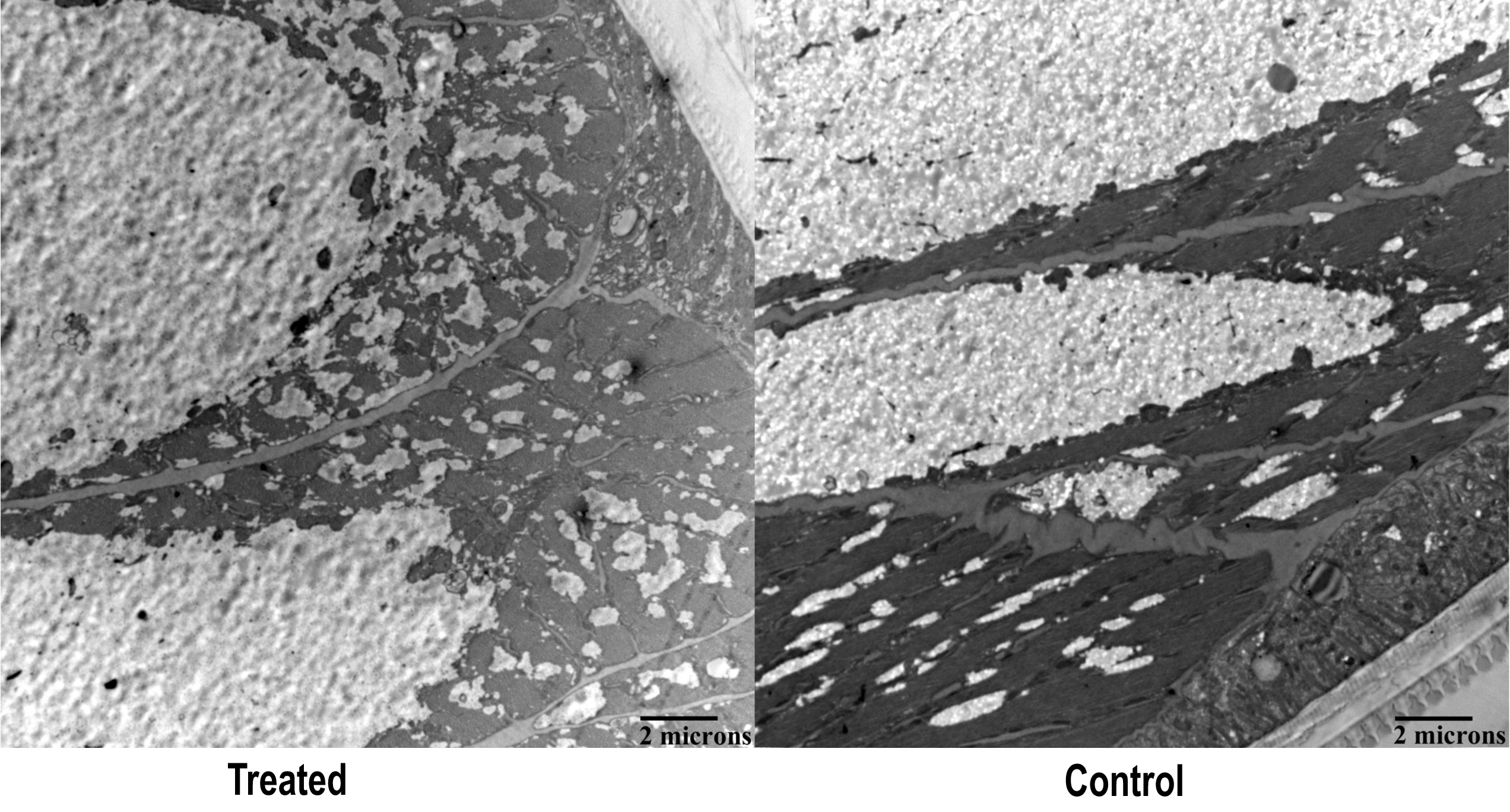

Supplement: S6 Fig — No definitive difference between treated and untreated worms. (TIF) [file pntd.0005690.s006.tif]

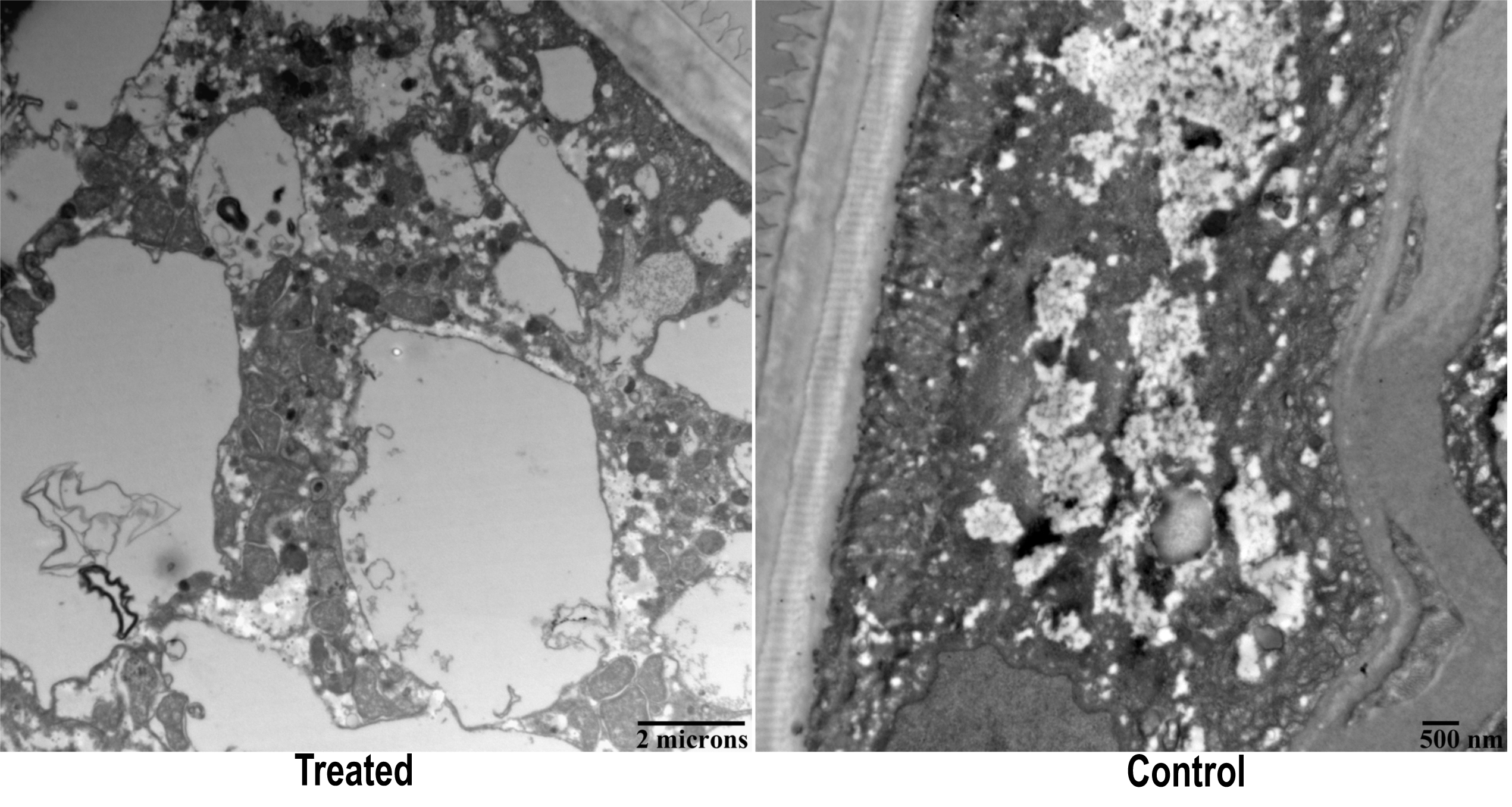

Supplement: S7 Fig — Diminished glycogen and distorted architecture in the treated worm. (TIF) [file pntd.0005690.s007.tif]

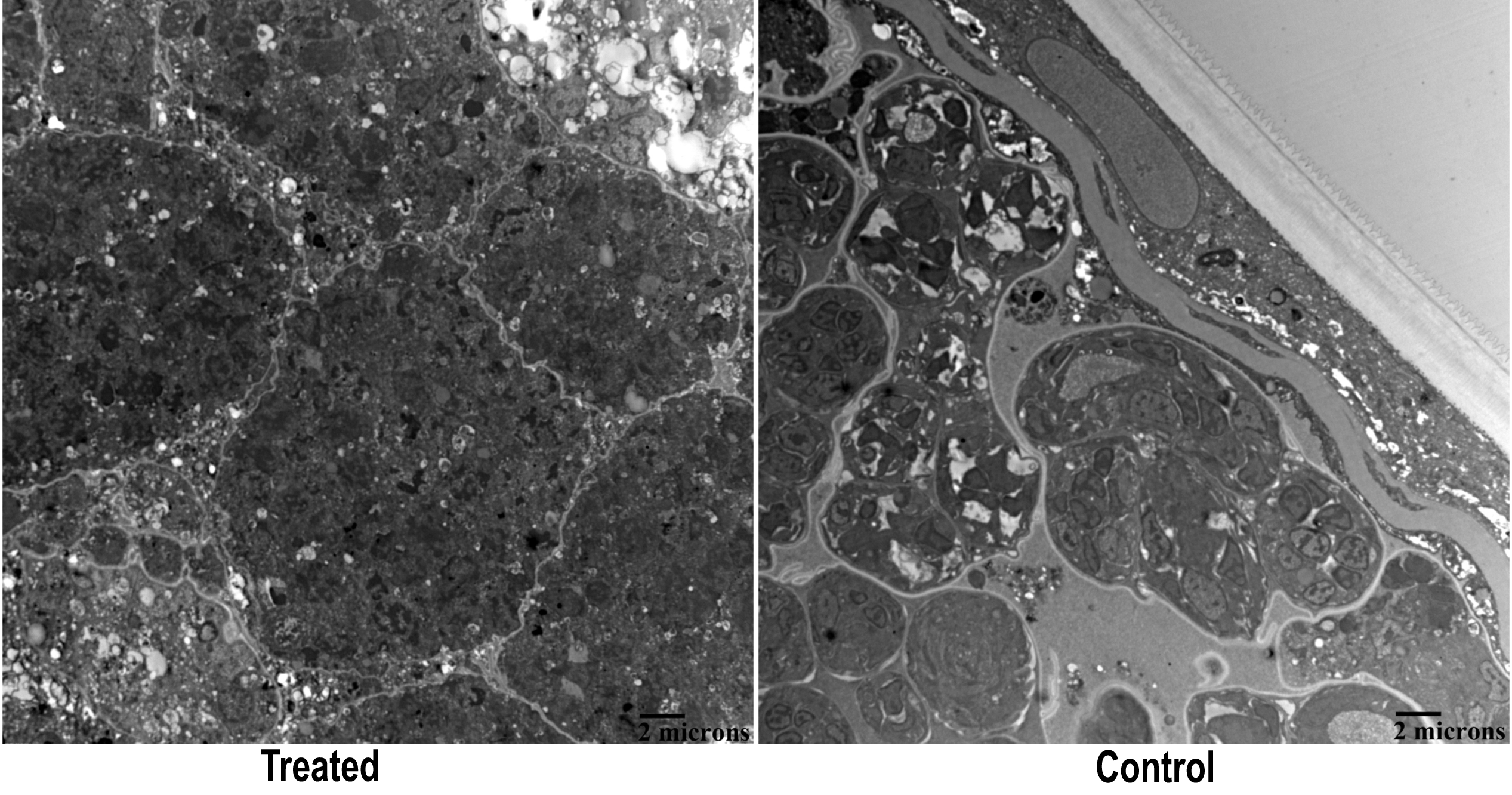

Supplement: S8 Fig — Damage beyond recognition of internal organelles of developing microfilariae in treatment group with all microfilariae being homogenously affected. (TIF) [file pntd.0005690.s008.tif]

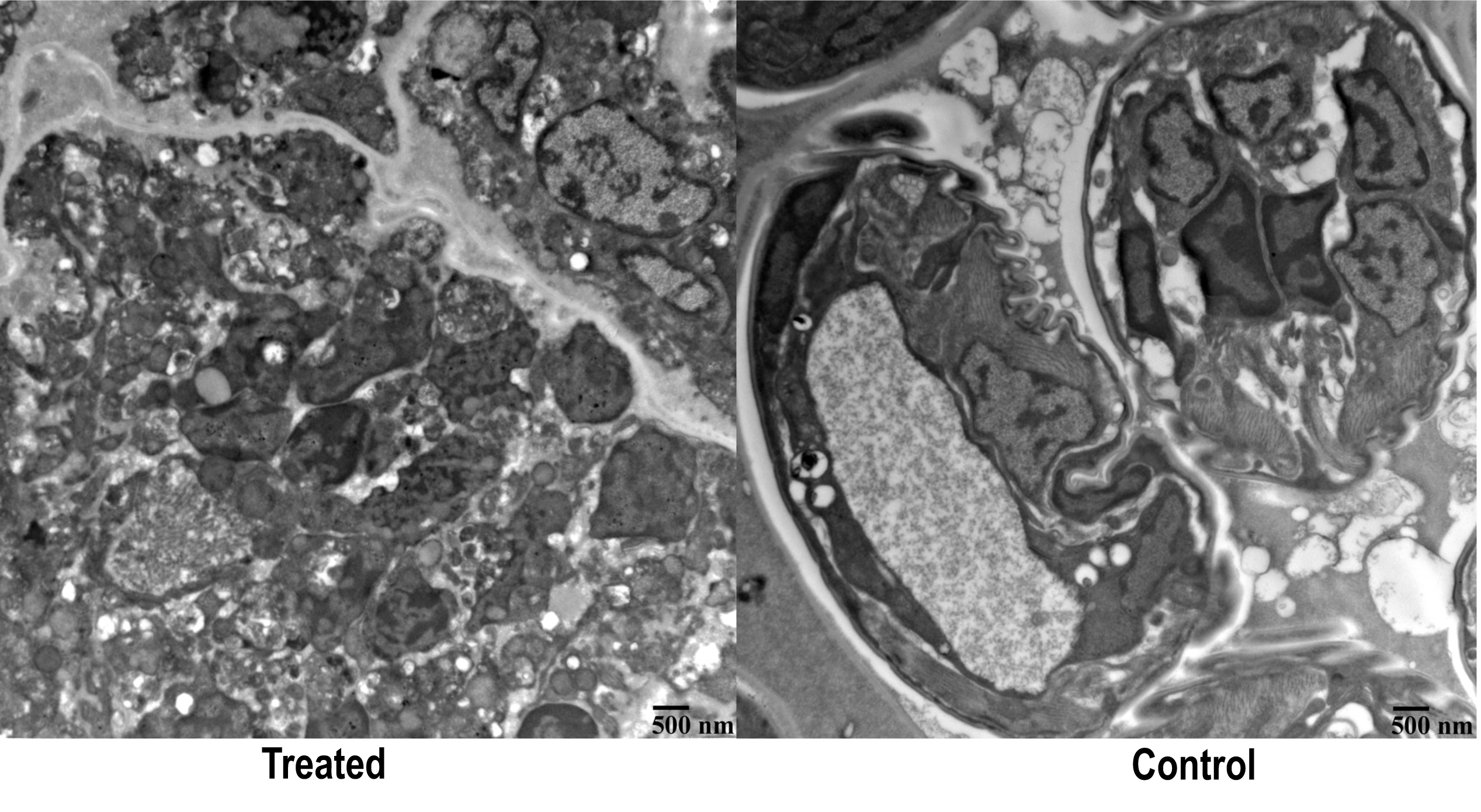

Supplement: S9 Fig — Internal structures of treated group not identifiable. (TIF) [file pntd.0005690.s009.tif]

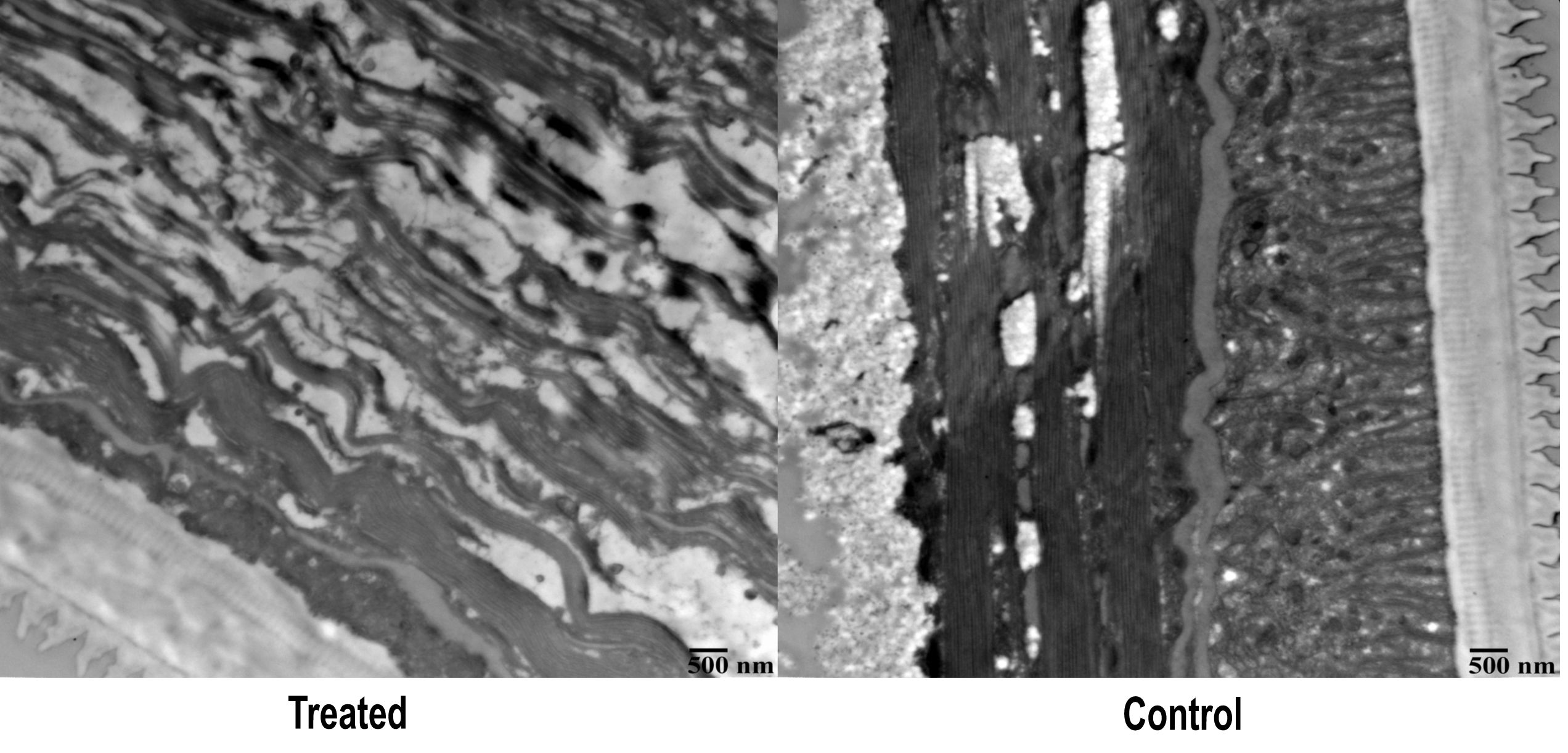

Supplement: S10 Fig — No significant changes. (TIF) [file pntd.0005690.s010.tif]

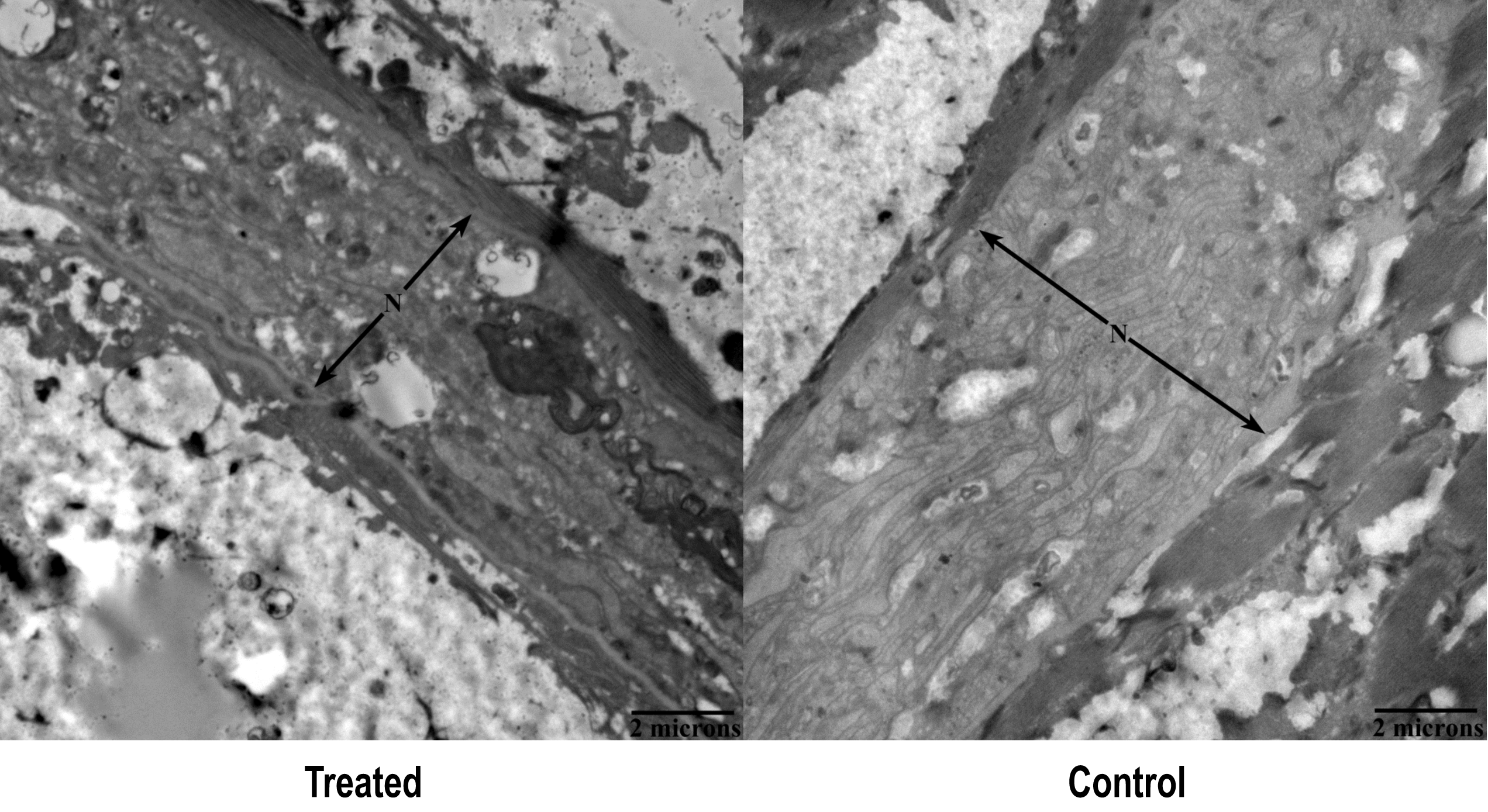

Supplement: S11 Fig — Treated worm with significant nerve damage. (TIF) [file pntd.0005690.s011.tif]

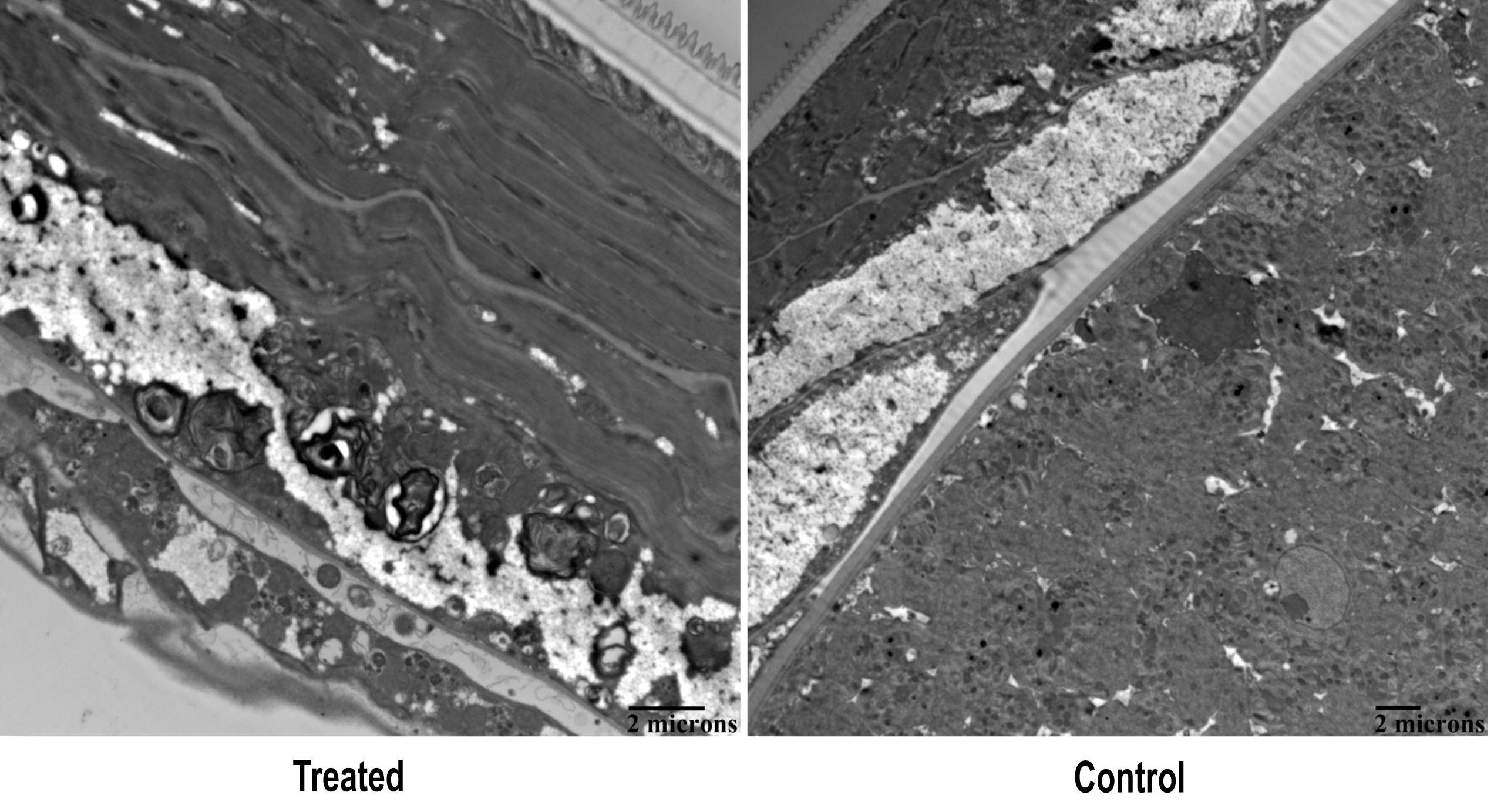

Supplement: S12 Fig — No obvious changes. (TIF) [file pntd.0005690.s012.tif]

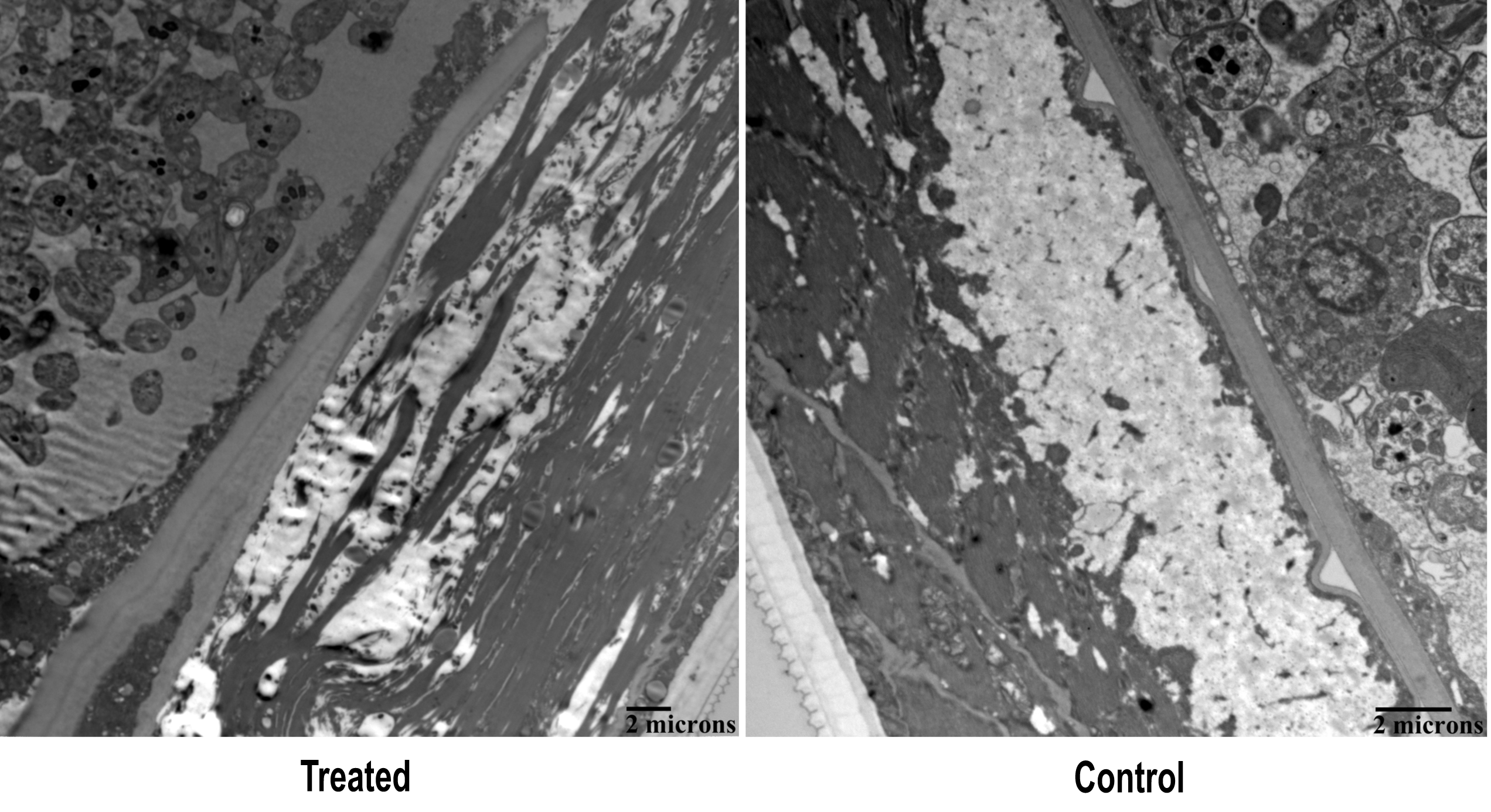

Supplement: S13 Fig — No definitive alterations of the muscle/glycogen organization. (TIF) [file pntd.0005690.s013.tif]

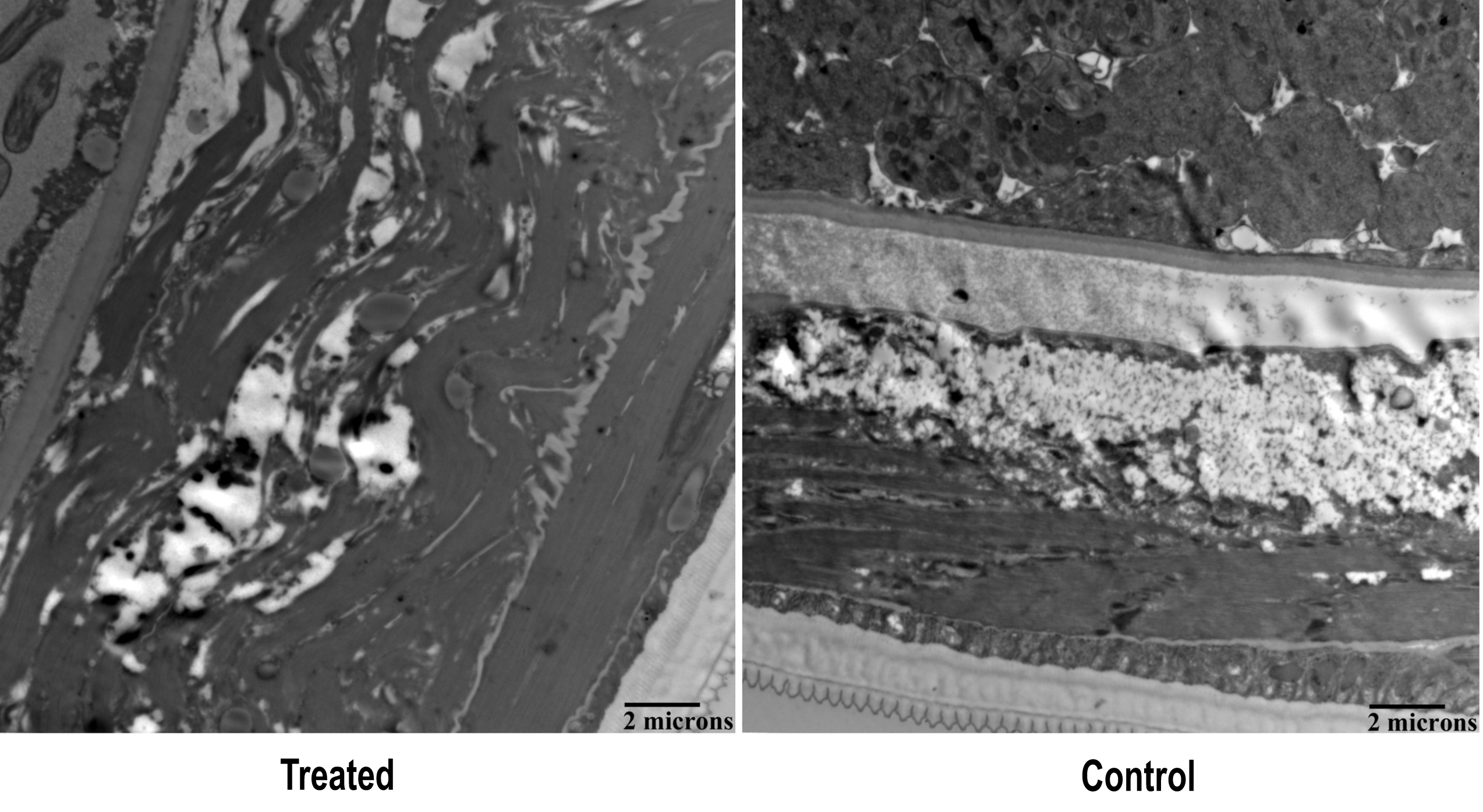

Supplement: S14 Fig — No definitive alterations of the muscle/glycogen organization. (TIF) [file pntd.0005690.s014.tif]

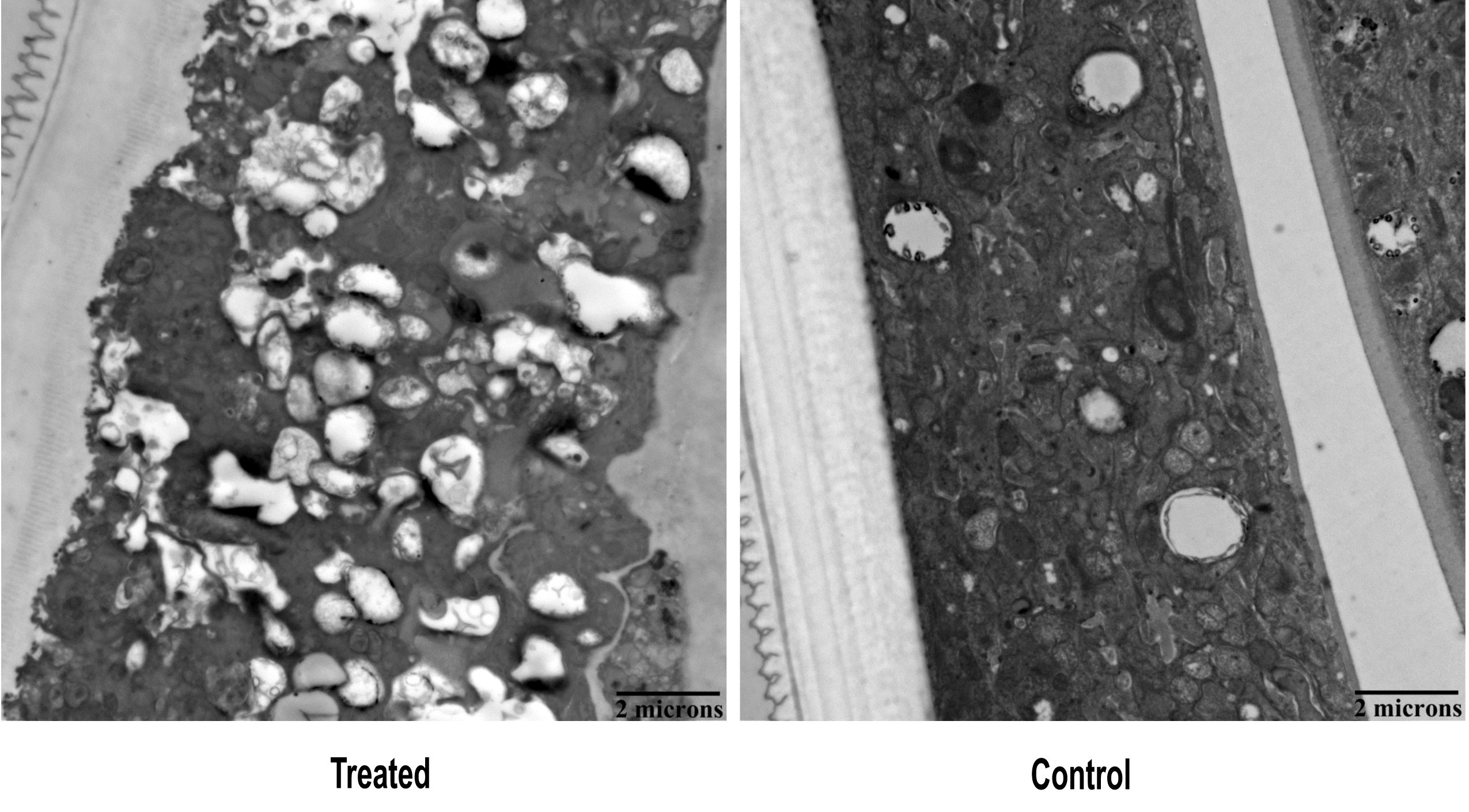

Supplement: S15 Fig — Alteration of hypodermal structures seen in treated worm. (TIF) [file pntd.0005690.s015.tif]

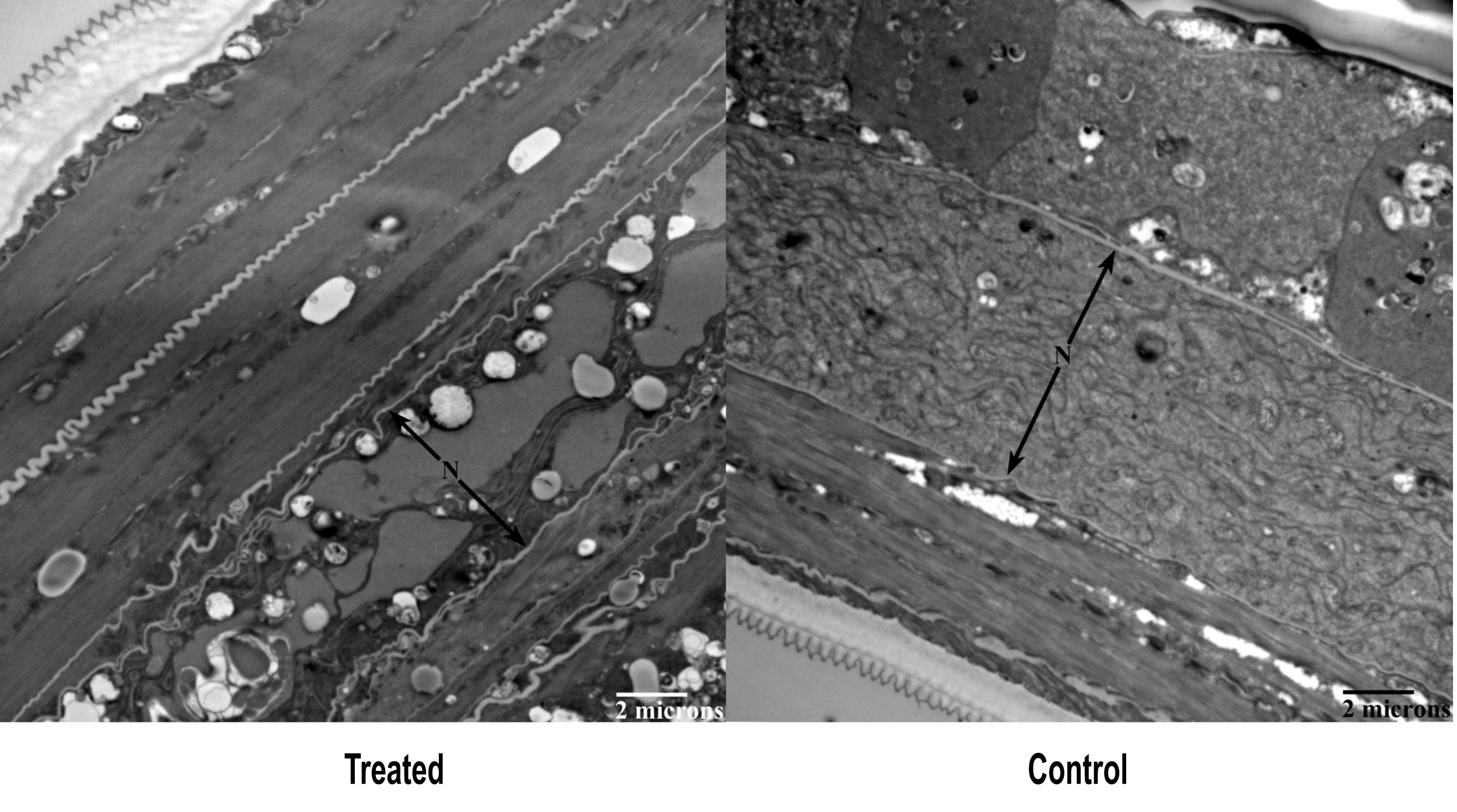

Supplement: S16 Fig — Treated worm with significant nerve damage. (TIF) [file pntd.0005690.s016.tif]

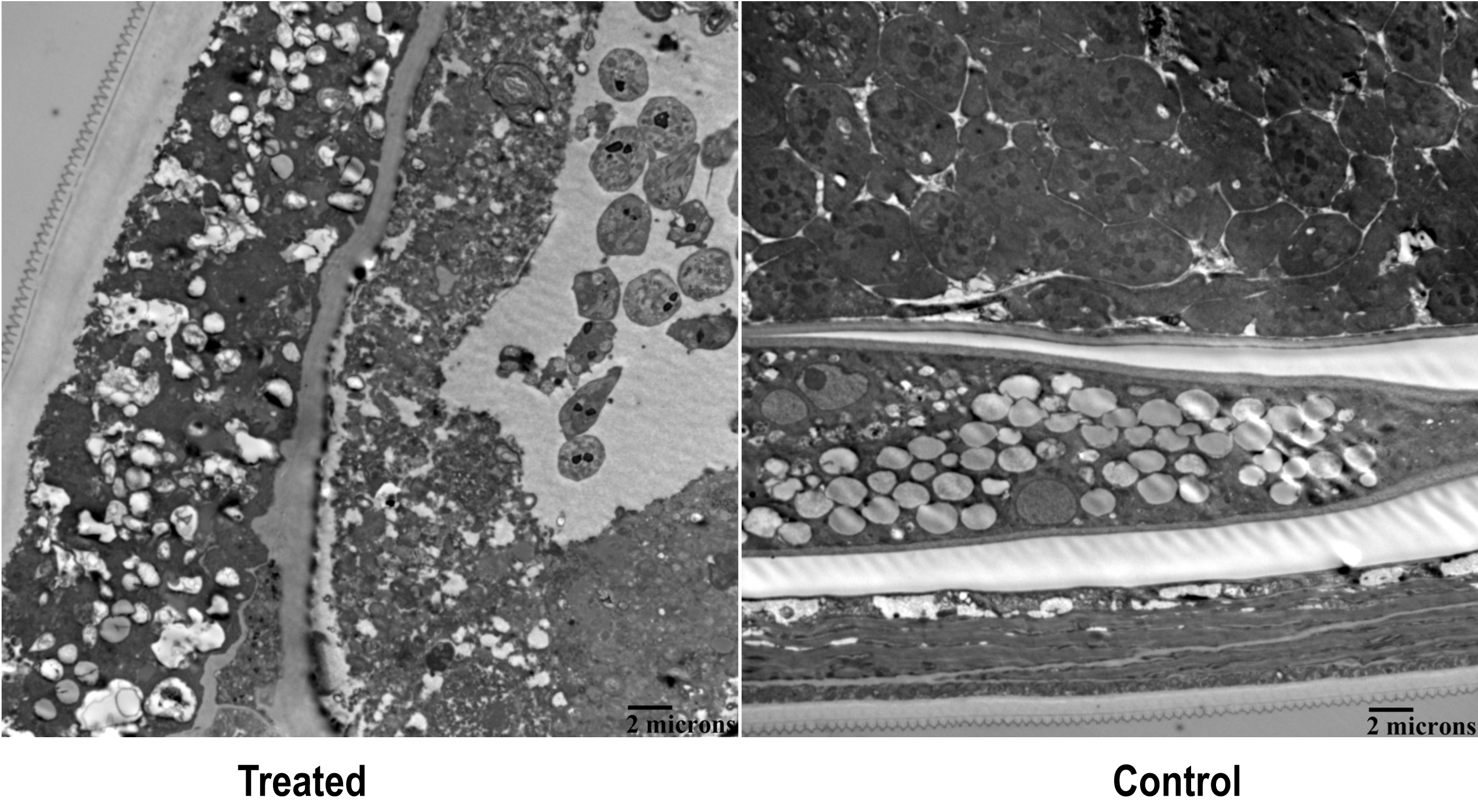

Supplement: S17 Fig — No clear changes. (TIF) [file pntd.0005690.s017.tif]

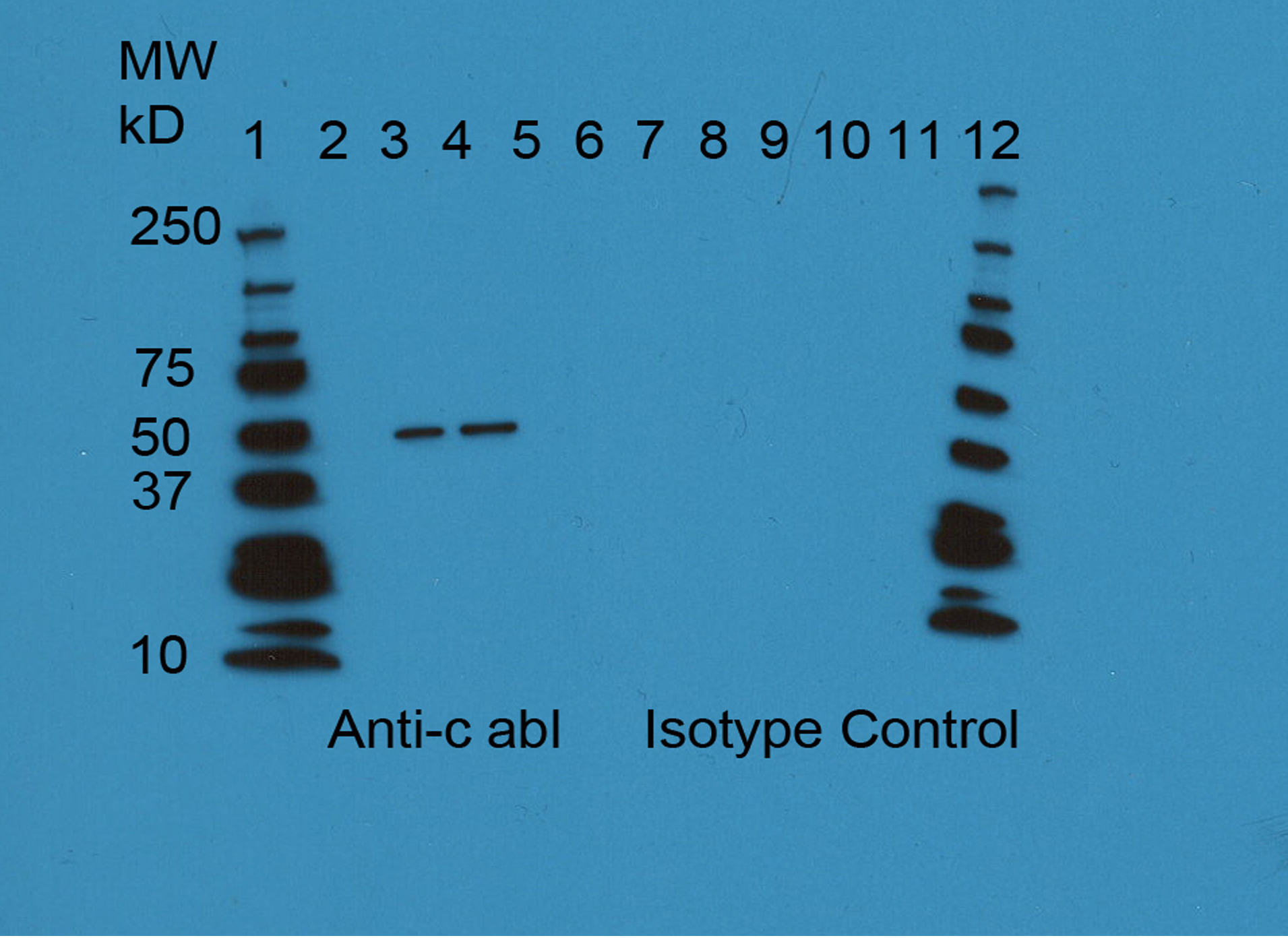

Supplement: S18 Fig — While no bands are present in the isotope control lanes, a single discrete band is observed in lanes 3 and 4, detected by the anti-c abl antibody and approximately 50kD in size. Molecular markers are in lanes 1 and 12. (TIF) [file pntd.0005690.s018.tif]
